# *Mycobacterium tuberculosis* Strains Potentially Involved in the TB Epidemic in Sweden a Century Ago

**DOI:** 10.1371/journal.pone.0046848

**Published:** 2012-10-08

**Authors:** Ramona Groenheit, Solomon Ghebremichael, Alexandra Pennhag, Jerker Jonsson, Sven Hoffner, David Couvin, Tuija Koivula, Nalin Rastogi, Gunilla Källenius

**Affiliations:** 1 Department of Preparedness, Swedish Institute for Communicable Disease Control, Solna, Sweden; 2 Tuberculosis and Mycobacteria Unit, WHO Supranational TB Reference Laboratory, Institut Pasteur de la Guadeloupe, Guadeloupe, France; 3 Department of Clinical Science and Education, Karolinska Institutet, Södersjukhuset, Stockholm, Sweden; Institut Pasteur de Lille, France

## Abstract

A hundred years ago the prevalence of tuberculosis (TB) in Sweden was one of the highest in the world. In this study we conducted a population-based search for distinct strains of *Mycobacterium tuberculosis* complex isolated from patients born in Sweden before 1945. Many of these isolates represent the *M. tuberculosis* complex population that fueled the TB epidemic in Sweden during the first half of the 20^th^ century.

**Methods:**

Genetic relationships between strains that caused the epidemic and present day strains were studied by spoligotyping and restriction fragment length polymorphism.

**Results:**

The majority of the isolates from the elderly population were evolutionary recent Principal Genetic Group (PGG)2/3 strains (363/409 or 88.8%), and only a low proportion were ancient PGG1 strains (24/409 or 5.9%). Twenty-two were undefined. The isolates demonstrated a population where the Euro-American superlineage dominated; in particular with Haarlem (41.1%) and T (37.7%) spoligotypes and only 21.2% belonged to other spoligotype families. Isolates from the elderly population clustered much less frequently than did isolates from a young control group population.

**Conclusions:**

A closely knit pool of PGG2/3 strains restricted to Sweden and its immediate neighbours appears to have played a role in the epidemic, while PGG1 strains are usually linked to migrants in todaýs Sweden. Further studies of these outbreak strains may give indications of why the epidemic waned.

## Introduction

Tuberculosis (TB) is globally a major cause of morbidity and mortality, with a majority of cases occurring in low income countries. As estimated by the WHO currently one third of the world´s population is infected with bacteria of the *Mycobacterium tuberculosis* complex, and ten million cases of active TB disease occur each year, resulting in almost two million deaths annually. The increasing spread of TB has also been paralleled by a rapid increase in multi-drug resistant TB in many parts of the world, making the disease in several instances practically incurable.

Distinct *M. tuberculosis* strains have been associated with large outbreaks of TB. Also in the Nordic countries there are outbreaks of specific strains of *M. tuberculosis*. During the last decades, a specific strain of *M. tuberculosis* has emerged rapidly in Denmark [1], another outbreak has been recorded in Norway [2] and one of the largest outbreaks ever recorded in a low endemic country is ongoing in Sweden [3], [4], [5]. Little is known about the *M. tuberculosis* population that dominated Sweden a hundred years ago. It does however appear that this bacterial population has been successfully reduced from representing the major public health problem to its current level of near elimination.

Today the Nordic countries are high-income nations with low prevalence of TB. In 2011, the TB incidence in Sweden was 6.3/100,000 population and only 11% of the TB patients were born in Sweden. The number of cases is almost entirely dependent on migration from countries with high TB incidence and the group of elderly Swedish born patients with reactivated TB infection is decreasing [6]. However, less than a century ago the prevalence of TB in the Nordic countries (Denmark, Finland, Norway and Sweden) was among the highest in the world. In 1905 the overall estimated TB incidence in Sweden was 890/100,000 population [7] higher than in most high incidence countries of sub-Saharan Africa today.

Ten percent of otherwise healthy persons infected with *M. tuberculosis* are estimated to progress to active disease, with the highest risk during the first two years after infection. A combination of bacterial and patient factors influence the risk for induction of active disease in patients with latent infection, and many who were infected may develop TB after decades of infection [8]. In the elderly population in Sweden many are still latently infected with TB, and some develop active TB. In the cohorts born before 1945 most subjects presumably have latent TB infection (LTBI). Using a TB incidence among newborns of <1/100,000 population as an indicator of interrupted TB transmission must be interpreted with caution but by doing so one can estimate that since around 1967 [9], [10] most active TB cases occurring in the elderly Swedish-born population can be seen as reactivation of LTBI.

In the past decades our understanding of the molecular genetics of *M. tuberculosis* has further expanded. One of the major achievements using DNA fingerprinting techniques has been the implementation of population based transmission surveillance. Geographically defined lineages of *M. tuberculosis* have been identified [11] and specific genetically highly conserved groups of strains of *M. tuberculosis* have attracted special attention.

Worldwide, few collections of isolates from the 20^th^ century are available. At the Swedish Institute for Communicable Disease Control (SMI) clinical *M. tuberculosis* isolates have been preserved since the 1980s. This consequently provides us with an exceptional possibility to perform population-based studies of the transmission of TB. In this study we conduct the first systematic population-based search for distinct strains of *M. tuberculosis* isolated from elderly patients born in Sweden before 1945. The isolates represent strains most likely acquired in Sweden during the last 60–100 years, and many of these isolates may represent the *M. tuberculosis* population that fueled the TB epidemic in Sweden during the first half of the 20^th^ century.

## Materials and Methods

### Ethics Statement

At the SMI, clinical *M. tuberculosis* complex strains are routinely collected for disease surveillance. The current study describes a bacterial collection and the bacterial genotypes could only be combined with the sex, age, and country of birth for the patients from which the strains were isolated. Ethical approval was therefore not required.

### Bacterial Isolates

*M. tuberculosis* complex isolates obtained from all six Swedish TB laboratories in Gothenburg, Linköping, Malmö/Lund, Stockholm and Umeå during the years 1994–2009 were studied. They represent all strains from patients born in Sweden before 1945 that have been preserved at SMI during the sampling period and that did not cluster with any patients born after 1945 and/or were foreign born. During the same sampling period all isolates that had been preserved at SMI from patients born in Sweden in 1985 or later were also analysed as a control group. The patients were identified as born in Sweden through the national TB Register. The isolates had been stored at −70C°.

### Drug Susceptibility Testing

In Sweden, all isolates are tested for susceptibility to the first-line drugs isoniazid (INH), rifampicin (RIF), ethambutol (EMB) and pyrazinamide (PZA) using the BACTEC 460TB or the MGIT 960 liquid culture and drug susceptibility testing systems according to the instructions of the manufacturer. During the major part of the study all isolates were also tested for susceptibility to streptomycin (SM), except for the years 2004–2009, when the Linköping and Stockholm laboratories stopped testing for SM-resistance, since SM no longer was used for treatment of TB patients in Sweden. All laboratories had taken part in the external quality assurance program for drug susceptibility testing of *M. tuberculosis* offered by the Swedish TB reference laboratory at SMI.

### Spoligotyping

All isolates were characterized by spoligotyping, which characterizes the polymorphic direct repeat region of the *M. tuberculosis* chromosome [12]. The patterns obtained by spoligotyping were compared by visual examination and computer assisted analyses by use of the BioNumerics version 6.6 software (Applied Maths, Kortrijk, Belgium). The spoligotypes were also compared with those contained in the international database SITVIT2, an updated version of the previously published SpolDB4 database [11] (http://www.pasteur-guadeloupe.fr:8081/SITVITDemo) which defines 62 spoligotype families/subfamilies of *M. tuberculosis* complex isolates. The SITVIT2 database contains to date genotyping data on more than 86,000 clinical isolates from 160 countries of origin, with more than 3,000 spoligo-international-types (SITs; a pattern shared by two or more patient isolates). The BioNumerics software version 3.5 was used to build spoligotyping-based minimum spanning trees (MST). MST is an undirected network in which all of the samples are linked together with the fewest possible linkages between nearest neighbors. Using this approach, one considers that all intermediate stages are present within the sample analyzed by first including the individual that shows the greatest number of possible linkages to other individuals in the population studied. We used this method to highlight the links between the spoligotype families differing by changes observed in their direct variable repeats. To evaluate the distribution of orphan strains in order to get information on their specific evolution two trees were generated - one for SITs, and another for all patterns, i.e. SITs and orphans pooled together. We also created spoligoforest trees [13] to illustrate probable strain evolutionary relationships between spoligotypes. Contrarily to the MSTs the spoligoforest trees are directed and only evolve by loss of spacers. Lastly, the major *M. tuberculosis* genotypic families were also linked to “ancient” and “modern” lineages of tubercle bacilli as defined by Principal Genetic Groups (PGG) defined by *katG463-gyrA95* polymorphism [14], and inferred from the reported linking of specific spoligotype patterns to PGG1, 2 or 3 grouping [15], [16], [17], [18], [19]. For statistical analyses the isolates were categorised into three groups, modern [consisting of the Haarlem (H), T, Latin-American and Mediterranean (LAM), X and S families], ancient [consisting of Beijing, East-African-Indian (EAI) and Central-Asian (CAS) families] and [*M. bovis/M. bovis* like and Manu].

### IS*6110* RFLP

The isolates were cultured on Löwenstein Jensen medium, DNA was extracted and RFLP typing was performed using the insertion sequence IS*6110* as a probe and *Pvu*II as the restriction enzyme [20]. Visual bands were analyzed using the BioNumerics version 6.6 software. On the basis of the molecular sizes of the hybridizing fragments and the number of IS*6110* copies of each isolate, fingerprint patterns were compared by the un-weighted pair-group method of arithmetic averaging using the Jaccard coefficient. Dendrograms were constructed to show the degree of relatedness among strains according to a previously described algorithm [21] and similarity matrixes were generated to visualize the relatedness between the banding patterns of all isolates. The RFLP patterns were entered into the RFLP database at SMI, which at the time of this study contained 3951 isolates that had been isolated in Sweden. Strains with identical RFLP-patterns (100% similarity) were judged to belong to a cluster.

### Statistical Method

Mean and standard deviation (SD) were calculated for age of the patient at diagnosis and year of birth by isolate group. Age and year of birth were not normally distributed and therefore possible differences in these variables for isolate group and gender were investigated with the non-parametric Kruskal-Wallis test. Post-hoc tests for isolate group were performed for the three pairs using the Kruskal-Wallis test (Mann-Whitney U test). The chi^2^-test was used to test for association between two categorical variables. The level of significance was set to 0.05 (two-sided) and all analyses were performed using R v 2.9.2 (R Foundation for Statistical Computing, Vienna, Austria).

## Results

In total, 409 isolates from 242 (59.2%) men and 167 (40.8%) women born in Sweden before 1945 were analysed. These patients were born in Sweden between the years 1908–1945 (Table S1). A total of 9.8% were born before 1914, 19.6% were born in 1915–1919, 27.1% were born in 1920–1924, 17.1% were born in 1925–1929, 11.0% were born in 1930–1934, 7.3% were born in 1935–1939 and 8.1% were born in 1940–1945. At diagnosis the patients were 52–98 years old, with a mean age of 78.1 years. The 58 patients in the young Swedes control group were born between the years 1985–2008 (Tabel S2). A total of 24.1% were born in 1985–1989, 37.9% were born in 1990–1994, 17.2% were born in 1995–1999, 8.6% were born in 2000–2004 and 12.1% were born in 2005–2008. At diagnosis the patients were 0–23 years old, with a mean age of 10.9 years.

### Drug Resistance

Among the elderly Swedes, information on drug resistance was obtained for 404/409 isolates. Of those, 38 isolates (9.3%) were resistant to one or more of the drugs SM (n = 3), INH (n = 14), RIF (n = 4), EMB (n = 1) and PZA (n = 22). The large number of isolates resistant to PZA is explained by the inclusion of *M. bovis* isolates in the study. Five isolates were resistant to more than one drug, and of those four isolates were multidrug resistant. Among the 58 young Swedes in the control group, 17 isolates (29.3%) were resistant to one or more of the drugs SM (n = 5), INH (n = 16), RIF (n = 2), EMB (n = 1) and PZA (n = 1). Five isolates were resistant to more than one drug, and of those two were multidrug resistant. As some laboratories during the study period stopped testing for SM resistance, only 207/409 and 17/58 of the isolates were analysed for SM resistance.

### Spoligotyping

Out of 409 isolates, 173 different spoligo patterns were obtained, of which 277 (67.7%) were clustered in 41 spoligo clusters comprising 2–56 strains per cluster. The remaining 132 (32.3%) spoligo patterns were unique i.e. the isolates did not cluster with other patient isolates. When compared with SITVIT2, the majority, 364 clinical isolates, were shared-types (Table 1), i.e. had an identical pattern shared by two or more isolates worldwide (within this study, or matching another strain in the SITVIT2 database). A SIT number was attributed to each pattern according to the SITVIT2 database. Forty-five patterns corresponded to orphan strains that were unique among the 86,000 strains recorded in the SITVIT2 database (Table 2). The isolates demonstrated a highly homogenous population where the modern H and T clades dominated. The absolute majority (n = 363, 88.8%) were evolutionary recent PGG2/3 strains, including H (n = 168, 41.1%), T (n = 154, 37.7%), LAM (n = 32), S (n = 8) and X (n = 1) isolates (Table 1 and S3). Only 24 (5.9%) were evolutionary ancient PGG1 strains (3 Beijing, 3 CAS1-Delhi, 4 EAI, 3 Manu and 11 *M. bovis/M. bovis* like isolates) (Table 1 and S4). Twenty-two spoligotyping signatures that are not yet associated to a well-defined spoligotype familiy in SITVIT2 were designated as “Unknown” (Table 1 and 2). The most common spoligotypes were SIT50 (n = 56, 13.7%) of the H3 subfamily, SIT53 (n = 43, 10.5%) of the T1 subfamily, SIT47 (n = 33, 8.1%) of the H1 subfamily and SIT42 (n = 14, 3.4%) of the LAM9 subfamily (Table S3). In addition to the T1 subfamily prototype, SIT53, two more T clade SITs, SIT153 (n = 11, 2.7%) and SIT37 (n = 7, 1.7%) were among the seven predominant SITs. Four of the *M. bovis* isolates (SIT691) lacked spacer 11, in addition to spacers 3, 9, 16, and 39 to 43. Of the 45 orphan strains, 22 were of the T spoligotype family, 17 of the H family, 2 *M. bovis/M. bovis* like, 1 LAM family and 3 of unknown family. Significant for all except the two *M. bovis* strains was that they all lacked spacers 33–36 (signature of SIT53).

**Table 1:** Description of 127 shared-types (SITs; n = 364 isolates, 2–56 isolates per cluster) and corresponding spoligotyping families/subfamilies starting from a total of 409 *M. tuberculosis* complex strains isolated from Swedish patients born before 1945.

SIT*	Spoligotype Description	OctalNumber	Number(%) instudy	% in studyvs.Database	Spoligotypefamily**	Clustered vs.uniquepatterns***
1	□□□□□□□□□□□□□□□□□□□□□□□□□□□□□□□□□□▪▪▪▪▪▪▪▪▪	000000000003771	3 (0,73)	0,03	Beijing	Clustered
2	□□□□□□□□□□□□□□□□□□□□□□□□▪□□□□□□▪□□□□▪▪▪▪▪▪▪	000000004020771	1 (0,24)	0,25	H2	Unique
4	□□□□□□□□□□□□□□□□□□□□□□□□▪▪▪▪▪▪▪▪□□□□▪▪▪▪▪▪▪	000000007760771	1 (0,24)	0,29	Unknown	Unique
19	▪▪□▪▪▪▪▪▪▪▪▪▪▪▪▪▪▪▪□□▪▪▪▪▪▪▪□□□□▪□▪▪▪▪▪▪▪▪▪	677777477413771	3 (0,73)	0,35	EAI2-Manilla	Clustered
20	▪▪□▪▪▪▪▪▪▪▪▪▪▪▪▪▪▪▪▪□□□□▪▪▪▪▪▪▪▪□□□□▪▪▪▪▪▪▪	677777607760771	4 (0,98)	0,49	LAM1	Clustered
26	▪▪▪□□□□▪▪▪▪▪▪▪▪▪▪▪▪▪▪▪□□□□□□□□□□□□▪▪▪▪▪▪▪▪▪	703777740003771	1 (0,24)	0,08	CAS1-Delhi	Unique
29	▪▪▪▪▪□□□□□□□□□□□□▪▪□□□□□□□□□□□□□□□□□□□▪▪▪▪▪	760001400000171	1 (0,24)	2,44	LAM	Unique
32	▪▪▪▪▪▪▪▪□□□□□□□□□□□□□□□□□□□□□□□□□□□□□□▪▪▪▪▪	776000000000171	2 (0,49)	1,92	Unknown	Clustered
34	▪▪▪▪▪▪▪▪□□▪▪▪▪▪▪▪▪▪▪▪▪▪▪▪▪▪▪▪▪▪▪□□□□▪▪▪▪▪▪▪	776377777760771	6 (1,47)	0,73	S	Clustered
36	▪▪▪▪▪▪▪▪▪▪▪▪□▪▪▪▪▪▪▪▪▪▪▪▪▪▪▪▪▪□▪□□□□▪▪▪▪▪▪▪	777737777720771	3 (0,73)	2,34	H3	Clustered
37	▪▪▪▪▪▪▪▪▪▪▪▪□▪▪▪▪▪▪▪▪▪▪▪▪▪▪▪▪▪▪▪□□□□▪▪▪▪▪▪▪	777737777760771	7 (1,71)	1,55	T3	Clustered
39	▪▪▪▪▪▪▪▪▪▪▪▪▪▪▪▪▪▪□▪▪▪□□▪▪▪▪▪▪▪▪□□□□▪□□▪▪▪▪	777777347760471	1 (0,24)	0,71	T4-CEU1	Unique
40	▪▪▪▪▪▪▪▪▪▪▪▪▪▪▪▪▪▪□▪▪▪▪▪▪▪▪▪▪▪▪▪□□□□▪▪▪▪▪▪▪	777777377760771	1 (0,24)	0,65	T4	Unique
42	▪▪▪▪▪▪▪▪▪▪▪▪▪▪▪▪▪▪▪▪□□□□▪▪▪▪▪▪▪▪□□□□▪▪▪▪▪▪▪	777777607760771	14 (3,42)	0,44	LAM9	Clustered
44	▪▪▪▪▪▪▪▪▪▪▪▪▪▪▪▪▪▪▪▪▪▪□▪▪▪▪▪▪▪▪▪□□□□▪▪▪▪▪▪▪	777777757760771	1 (0,24)	0,48	T5	Unique
45	▪▪▪▪▪▪▪▪▪▪▪▪▪▪▪▪▪▪▪▪▪▪▪□▪□□□□□□▪□□□□▪▪▪▪▪▪▪	777777764020771	2 (0,49)	1,89	H1	Clustered
47	▪▪▪▪▪▪▪▪▪▪▪▪▪▪▪▪▪▪▪▪▪▪▪▪▪□□□□□□▪□□□□▪▪▪▪▪▪▪	777777774020771	33 (8,07)	2,26	H1	Clustered
49	▪▪▪▪▪▪▪▪▪▪▪▪▪▪▪▪▪▪▪▪▪▪▪▪▪▪▪▪▪▪□▪□□□□▪▪▪□▪▪▪	777777777720731	12 (2,93)	6,98	H3	Clustered
50	▪▪▪▪▪▪▪▪▪▪▪▪▪▪▪▪▪▪▪▪▪▪▪▪▪▪▪▪▪▪□▪□□□□▪▪▪▪▪▪▪	777777777720771	56 (13,69)	1,71	H3	Clustered
51	▪▪▪▪▪▪▪▪▪▪▪▪▪▪▪▪▪▪▪▪▪▪▪▪▪▪▪▪▪▪▪▪□□□□▪▪▪□□□□	777777777760700	1 (0,24)	0,36	T1	Unique
52	▪▪▪▪▪▪▪▪▪▪▪▪▪▪▪▪▪▪▪▪▪▪▪▪▪▪▪▪▪▪▪▪□□□□▪▪▪□▪▪▪	777777777760731	3 (0,73)	0,33	T2	Clustered
53	▪▪▪▪▪▪▪▪▪▪▪▪▪▪▪▪▪▪▪▪▪▪▪▪▪▪▪▪▪▪▪▪□□□□▪▪▪▪▪▪▪	777777777760771	43 (10,51)	0,72	T1	Clustered
54	▪▪▪▪▪▪▪▪▪▪▪▪▪▪▪▪▪▪▪▪▪▪▪▪▪▪▪▪▪▪▪▪□□▪▪▪▪▪▪▪▪▪	777777777763771	1 (0,24)	0,45	MANU2	Unique
62	▪▪▪▪▪▪▪▪▪▪▪▪▪▪▪▪▪▪▪▪▪▪▪▪▪□□□□□□▪□□□□▪▪▪□▪▪▪	777777774020731	4 (0,98)	0,73	H1	Clustered
64	▪▪▪▪▪▪▪▪▪▪▪▪▪▪▪▪▪▪▪▪□□□□▪▪▪▪□▪▪▪□□□□▪▪▪▪▪▪▪	777777607560771	2 (0,49)	0,56	LAM6	Clustered
65	▪▪▪▪▪▪▪▪▪▪▪▪▪▪▪▪▪▪▪▪▪▪▪▪▪▪▪▪▪▪▪▪□□□□▪□□▪▪▪▪	777777777760471	1 (0,24)	0,95	T1	Unique
78	▪▪▪▪▪▪▪▪▪▪▪▪▪▪▪▪▪▪▪▪▪▪▪▪▪▪▪▪▪▪▪▪□□□□▪▪▪□□▪▪	777777777760711	1 (0,24)	1,56	T	Unique
97	▪▪▪▪▪▪▪▪▪▪▪▪▪▪▪▪▪▪▪▪▪▪▪□□□□▪▪▪▪▪□□□□▪▪▪▪▪▪▪	777777760760771	1 (0,24)	14,29	T1	Unique
99	▪▪▪▪□▪▪▪▪▪▪▪▪▪▪▪▪▪▪▪▪▪▪▪▪▪▪▪▪▪□▪□□□□▪▪▪▪▪▪▪	757777777720771	1 (0,24)	1,33	H3	Unique
100	▪▪▪▪▪▪▪▪▪▪▪▪▪▪▪▪▪▪▪▪▪▪▪▪▪▪▪▪▪▪▪▪▪□▪▪▪▪▪▪▪▪▪	777777777773771	1 (0,24)	1,32	MANU1	Unique
123	▪▪▪▪▪▪▪▪▪▪▪▪▪▪▪▪▪▪▪▪▪▪▪▪▪▪□□▪▪▪▪□□□□▪▪▪▪▪▪▪	777777776360771	1 (0,24)	4,17	T1	Unique
134	▪▪▪▪▪▪▪▪▪▪▪▪▪▪▪▪▪▪▪▪▪▪▪▪▪▪▪▪▪▪□▪□□□□▪▪□□▪▪▪	777777777720631	1 (0,24)	1,92	H3	Unique
151	▪▪▪▪▪▪▪▪▪▪▪▪▪▪□▪▪▪▪▪▪▪▪▪▪□□□□□□▪□□□□▪▪▪▪▪▪▪	777767774020771	1 (0,24)	4	H1	Unique
153	▪▪▪▪□▪▪▪▪▪▪▪▪▪▪▪▪▪▪▪▪▪▪▪▪▪▪▪▪▪▪▪□□□□▪▪▪□▪▪▪	757777777760731	11 (2,69)	14,86	T2	Clustered
167	▪▪▪▪▪▪▪▪▪▪▪▪▪▪▪▪▪▪▪▪▪▪▪▪▪▪▪▪▪□▪▪□□□□▪▪▪▪▪▪▪	777777777660771	3 (0,73)	4,17	T1	Clustered
172	▪▪▪▪▪▪▪▪▪▪▪▪▪▪▪▪▪▪▪▪▪▪▪▪▪▪▪▪▪▪▪□□□□□▪▪▪▪▪▪▪	777777777740771	1 (0,24)	1,82	T1	Unique
183	▪▪▪▪▪▪▪▪▪▪▪▪▪▪▪▪▪▪□▪▪▪▪▪▪▪▪▪▪▪□▪□□□□▪▪▪▪▪▪▪	777777377720771	1 (0,24)	1,85	H3	Unique
191	□□▪▪▪▪▪▪▪▪▪▪▪▪▪▪▪▪▪▪▪▪▪▪▪▪▪▪▪▪▪▪□□□□▪▪▪▪▪▪▪	177777777760771	1 (0,24)	5,56	T1	Unique
205	▪▪▪□▪▪▪▪▪▪▪▪▪▪▪▪▪▪▪▪▪▪▪▪▪▪▪▪▪▪▪▪□□□□▪▪▪▪▪▪▪	737777777760771	1 (0,24)	1,92	T1	Unique
209	▪▪▪▪▪▪▪▪□□□□□□▪▪▪▪▪▪□□□□▪▪▪▪▪▪▪▪□□□□▪▪▪▪▪▪▪	776017607760771	1 (0,24)	2,44	LAM12-Madrid1	Unique
218	▪▪▪▪▪▪▪▪▪▪▪▪□▪▪▪▪▪▪▪▪▪▪▪▪□□□□□□▪□□□□▪▪▪▪▪▪▪	777737774020771	3 (0,73)	8,33	H1	Clustered
241	▪▪▪▪▪▪▪▪▪▪▪▪▪▪▪▪▪▪▪▪▪▪▪▪▪▪▪▪▪▪▪▪□□□□▪□□□□▪▪	777777777760411	5 (1,22)	21,74	T1	Clustered
254	▪▪▪▪▪▪▪▪▪▪▪▪▪▪□□□□□□□□□□▪▪▪▪▪▪▪▪□□□□▪▪▪▪▪▪▪	777760007760771	1 (0,24)	0,6	T5-RUS1	Unique
262	▪▪▪▪▪▪▪□□▪▪▪▪▪▪▪▪▪▪▪▪▪▪▪▪▪▪▪□□□▪□□□□▪▪▪▪▪▪▪	774777777420771	1 (0,24)	0,57	H3	Unique
276	▪▪▪▪▪▪▪▪▪▪▪▪▪▪▪▪▪▪▪▪▪▪▪▪□□□□□□▪▪□□□□▪▪▪▪▪▪▪	777777770060771	1 (0,24)	2,78	T1	Unique
280	▪▪▪▪▪▪□□□□□□□□□□□□▪▪▪▪▪▪▪▪▪▪▪▪▪▪□□□□▪▪▪▪▪▪▪	770000777760771	2 (0,49)	2,94	T1-RUS2	Clustered
283	▪▪▪▪▪▪▪▪▪▪▪▪▪▪▪▪▪▪▪▪▪□□□▪□□□□□□▪□□□□▪▪▪▪▪▪▪	777777704020771	1 (0,24)	1,64	H1	Unique
353	▪▪▪▪▪▪▪▪▪▪▪▪▪▪▪▪▪▪▪▪▪▪▪▪▪□□▪▪▪▪▪□□□□▪▪▪▪▪▪▪	777777774760771	2 (0,49)	7,41	T1	Clustered
356	▪▪▪□□□□▪▪▪▪▪▪▪▪▪▪▪▪▪□□□□□□□□□□□□□□□▪▪▪▪▪▪▪▪	703777600001771	1 (0,24)	7,14	CAS1-Delhi	Unique
373	▪▪▪▪▪▪▪▪▪▪▪▪▪▪▪▪▪▪▪▪▪▪▪□▪▪▪▪▪▪▪▪□□□□▪▪▪▪▪▪▪	777777767760771	1 (0,24)	1,52	T1	Unique
383	▪▪▪▪▪▪▪▪□□▪▪▪▪▪▪▪▪▪▪▪▪▪▪▪□□□□□□▪□□□□▪▪▪▪▪▪▪	776377774020771	1 (0,24)	7,14	H1	Unique
393	▪▪▪▪▪▪▪▪▪▪▪▪▪□▪▪▪▪▪▪▪▪▪▪▪▪▪▪▪▪▪▪□□□□▪▪▪▪▪▪▪	777757777760771	2 (0,49)	5,56	T1	Clustered
452	▪▪▪▪▪▪▪▪▪▪▪▪▪▪▪▪▪▪▪▪□□□□▪▪□□▪▪▪▪□□□□▪▪▪▪▪▪▪	777777606360771	1 (0,24)	6,25	LAM9	Unique
453	▪▪▪▪▪▪▪▪▪▪▪▪▪▪▪▪▪▪▪▪□▪▪▪▪▪▪▪□▪▪▪□□□□▪▪▪▪▪▪▪	777777677560771	1 (0,24)	6,25	T1	Unique
463	▪▪▪▪▪▪▪▪▪▪▪▪▪▪▪▪▪▪▪▪▪▪▪▪▪▪▪▪▪▪□▪□□□□▪□▪▪▪▪▪	777777777720571	2 (0,49)	11,76	H3	Clustered
485	▪▪▪□□□□▪▪▪▪▪▪▪▪▪▪▪▪□□□□□□□□□□□□□□□▪▪▪▪▪▪▪▪▪	703777400003771	1 (0,24)	4,35	CAS1-Delhi	Unique
511	▪▪▪▪▪▪▪▪▪▪▪▪▪▪▪▪▪▪▪▪▪□□□□□□□□□□▪□□□□▪▪▪▪▪▪▪	777777700020771	4 (0,98)	9,09	H3	Clustered
515	▪▪▪▪▪▪▪▪▪▪▪▪▪▪▪▪▪▪▪▪▪▪▪▪▪▪□▪▪▪▪▪□□□□▪▪▪□▪▪▪	777777776760731	1 (0,24)	7,14	T2	Unique
523	▪▪▪▪▪▪▪▪▪▪▪▪▪▪▪▪▪▪▪▪▪▪▪▪▪▪▪▪▪▪▪▪▪▪▪▪▪▪▪▪▪▪▪	777777777777771	1 (0,24)	2,27	MANU_ancestor	Unique
524	▪▪▪▪▪▪▪▪▪▪▪▪▪▪▪▪▪▪▪▪▪▪▪▪▪▪▪▪▪▪□▪□□□□▪▪▪□□▪▪	777777777720711	1 (0,24)	8,33	H3	Unique
533	▪▪▪▪▪▪▪▪▪▪□□□□□□□□▪▪▪▪▪▪▪▪▪▪▪▪□▪□□□□▪▪▪▪▪▪▪	777400777720771	1 (0,24)	33,33	H3	Unique
602	▪▪▪▪▪▪▪▪▪▪▪▪▪▪▪▪▪▪▪▪▪▪▪▪□□□□□□□□□□□□▪▪▪▪▪▪▪	777777770000771	2 (0,49)	1,96	Unknown	Clustered
612	▪▪▪▪▪▪▪▪▪▪▪▪▪▪▪▪▪▪▪▪▪▪▪▪▪▪▪▪▪▪▪▪□□□□▪▪▪▪□▪▪	777777777760751	2 (0,49)	7,41	T1	Clustered
620	▪▪▪▪▪▪▪▪▪▪▪▪▪▪▪▪▪▪▪▪▪▪□□▪□□□□□□▪□□□□▪▪▪▪▪▪▪	777777744020771	1 (0,24)	7,14	H1	Unique
628	▪▪▪▪▪▪▪▪▪▪▪▪▪▪▪▪▪▪▪▪▪▪▪▪▪▪▪▪▪▪▪▪□□□□▪▪▪▪▪□□	777777777760760	1 (0,24)	6,67	T1	Unique
649	□▪▪▪▪▪▪▪▪▪▪▪▪▪▪▪▪▪▪□□□▪□□▪▪▪□▪▪▪□□□□▪▪▪▪▪▪▪	377777423560771	1 (0,24)	5,26	T1	Unique
691	▪▪□▪▪▪▪▪□▪□▪▪▪▪□▪▪▪▪▪▪▪▪▪▪▪▪▪▪▪▪▪▪▪▪▪▪□□□□□	676573777777600	4 (0,98)	0,55	BOV_1	Clustered
732	▪▪▪▪▪▪▪▪▪▪▪▪▪▪□□▪▪▪▪▪▪▪▪▪▪▪▪▪▪▪▪□□□□▪▪▪▪▪▪▪	777763777760771	1 (0,24)	4,17	T1	Unique
748	▪▪▪▪▪▪▪▪▪▪▪▪▪▪▪▪▪▪▪▪▪▪▪▪▪▪▪▪▪▪□▪□□□□▪▪▪▪▪□□	777777777720760	1 (0,24)	20	H3	Unique
766	▪▪▪▪▪▪▪▪▪▪▪▪▪▪□□□▪□□□□□□▪▪▪▪▪▪▪▪□□□□▪▪▪▪▪▪▪	777761007760771	2 (0,49)	4,17	LAM9	Clustered
781	□□□□□□□□□□□□□□□□□□□□□□□□▪□□□□□□▪□□□□▪▪▪□▪▪▪	000000004020731	1 (0,24)	7,69	H2	Unique
801	▪▪▪▪▪▪▪□□▪▪▪▪▪▪▪▪▪▪▪▪▪▪▪▪▪▪▪▪▪▪▪□□□□▪▪▪▪▪▪▪	774777777760771	1 (0,24)	11,11	T1	Unique
837	▪▪▪▪▪▪▪▪▪▪▪▪▪▪▪▪▪▪▪▪▪▪▪▪▪▪▪□□□□▪□□□□▪▪▪□▪▪▪	777777777020731	1 (0,24)	33,33	H	Unique
848	▪▪▪□▪▪▪▪▪▪▪▪▪▪▪▪▪▪▪▪▪▪▪▪▪▪▪▪▪▪▪▪□□□□▪▪▪□▪▪▪	737777777760731	4 (0,98)	13,79	T2	Clustered
888	▪▪▪▪▪▪▪▪▪▪▪▪▪▪▪▪▪▪▪▪▪▪▪▪▪▪▪▪▪▪▪▪□□□□▪▪□□▪▪▪	777777777760631	1 (0,24)	6,25	T1	Unique
913	▪▪▪▪▪▪▪▪▪▪▪▪▪□□□▪▪▪▪▪▪▪▪▪▪▪▪▪▪▪▪□□□□▪▪▪▪▪▪▪	777743777760771	1 (0,24)	5	T1	Unique
917	▪▪▪▪▪▪▪▪▪▪□▪▪▪▪▪▪▪▪▪▪▪▪▪▪▪▪▪▪▪▪▪□□□□▪▪▪▪▪▪▪	777577777760771	1 (0,24)	10	T1	Unique
918	▪▪▪▪▪▪□□□□□□□□□□□□▪▪□□□□▪▪▪▪▪▪▪▪□□□□▪▪▪▪▪▪▪	770000607760771	4 (0,98)	22,22	Unknown	Clustered
921	▪□□□▪▪▪▪▪▪▪▪□▪▪▪▪▪▪▪▪▪▪▪▪▪▪▪□□□▪□□□□▪▪▪▪▪▪▪	437737777420771	1 (0,24)	14,29	H4	Unique
926	▪▪▪▪▪▪□▪▪▪▪▪▪▪▪▪▪▪▪▪▪▪▪▪▪▪▪▪▪▪▪▪□□□□▪▪▪▪▪▪▪	773777777760771	1 (0,24)	3,13	T1	Unique
928	▪▪▪▪▪▪▪▪▪▪▪▪▪▪▪▪▪▪▪▪▪▪□▪□□□□□□▪▪□□□□▪▪▪▪▪▪▪	777777750060771	7 (1,71)	35	T	Clustered
946	▪▪▪▪▪▪▪▪▪▪▪▪▪▪▪▪▪▪▪▪▪▪□□□□□□□□□▪□□□□▪▪▪▪▪▪▪	777777740020771	1 (0,24)	5	H	Unique
947	▪▪▪▪▪▪▪▪▪▪▪▪▪▪▪▪▪▪▪▪▪▪▪▪▪▪▪▪□□□□▪□▪▪▪▪□□▪▪▪	777777777413631	1 (0,24)	10	EAI5	Unique
963	▪▪▪▪▪▪▪▪▪▪▪▪▪▪▪▪▪▪▪▪▪□▪▪▪▪▪▪▪▪□▪□□□□▪▪▪▪▪▪▪	777777737720771	1 (0,24)	8,33	H3	Unique
964	▪□□□▪▪▪▪▪▪▪▪▪▪▪▪▪▪▪▪□□□□▪▪▪▪▪▪▪▪□□□□▪▪▪▪▪▪▪	437777607760771	4 (0,98)	19,05	LAM1	Clustered
1107	▪▪▪▪▪▪▪▪▪▪▪▪□□□□□□□□□□□□▪▪▪▪▪▪▪▪□□□□▪▪▪▪▪▪▪	777700007760771	1 (0,24)	20	LAM	Unique
1173	▪▪▪▪▪▪□□□□□□□□□□□□▪▪▪▪▪▪▪▪▪▪▪▪▪▪□□□□▪▪▪□▪▪▪	770000777760731	1 (0,24)	14,29	T1-RUS2	Unique
1185	▪▪□▪▪▪▪▪□▪▪□▪▪▪□▪▪▪▪▪▪▪▪▪▪▪▪▪▪▪▪▪▪▪▪▪▪□□□□□	676673777777600	1 (0,24)	33,33	BOV_1	Unique
1211	▪□▪▪▪▪▪▪□□▪▪▪▪▪▪▪▪▪▪▪▪▪▪▪▪▪▪▪▪▪▪□□□□▪▪▪▪▪▪▪	576377777760771	1 (0,24)	5,56	S	Unique
1560	▪▪▪□□□□▪▪▪▪▪▪▪▪▪▪▪▪▪▪▪▪▪▪▪▪▪▪▪▪▪□□□□▪▪▪▪▪▪▪	703777777760771	1 (0,24)	14,29	T1	Unique
1561	▪▪▪▪▪▪▪▪▪▪▪▪▪▪▪▪▪▪▪▪▪▪▪▪▪□□□□□□▪□□□□□▪▪▪▪▪▪	777777774020371	1 (0,24)	25	H1	Unique
1611	▪▪▪▪▪▪▪▪□▪▪▪▪▪▪▪▪▪▪▪▪▪▪▪▪▪▪▪▪▪□▪□□□□▪▪▪▪▪▪▪	776777777720771	1 (0,24)	12,5	H3	Unique
1621	▪▪▪□▪▪▪▪▪▪▪▪□▪▪▪▪▪▪▪▪▪▪▪▪▪▪▪▪▪▪▪□□□□▪▪▪□▪▪▪	737737777760731	1 (0,24)	20	T	Unique
1626	▪▪▪▪▪▪▪▪▪▪▪▪▪▪▪▪▪▪▪▪▪▪▪▪▪▪□▪▪▪▪▪□□□□▪▪▪▪▪▪▪	777777776760771	2 (0,49)	16,67	T1	Clustered
1807	▪▪▪▪▪▪▪▪▪▪▪▪▪▪▪▪▪▪▪▪▪▪▪▪▪□□□□□□▪□□□□▪□▪▪▪▪▪	777777774020571	1 (0,24)	11,11	H1	Unique
1915	▪▪▪▪▪▪▪▪□□▪▪▪▪▪▪▪▪▪▪▪□▪▪▪▪▪▪▪▪▪▪□□□□▪▪▪▪▪▪▪	776377737760771	1 (0,24)	25	S	Unique
2201	□□▪▪▪▪▪▪▪▪▪▪▪▪▪▪▪▪▪▪□□□□▪▪▪▪▪▪▪▪□□□□▪▪▪▪▪▪▪	177777607760771	1 (0,24)	12,5	LAM9	Unique
2644	□□▪▪▪▪▪▪▪▪▪▪▪▪▪▪▪▪▪▪▪▪▪▪▪▪▪▪▪▪□▪□□□□▪▪▪▪▪▪▪	177777777720771	1 (0,24)	14,29	H3	Unique
2721	▪▪▪▪▪▪▪▪▪▪▪▪▪▪▪▪▪▪▪▪▪□□□□□□□□□□□□□□□□□▪▪▪▪▪	777777700000171	1 (0,24)	25	Unknown	Unique
2912	▪▪▪▪▪▪▪▪▪▪▪▪▪▪▪▪▪▪□▪▪▪□▪□□□□□□▪▪□□□□▪▪▪▪▪▪▪	777777350060771	1 (0,24)	33,33	T	Unique
2954	▪▪□▪▪▪▪▪▪▪▪▪▪▪▪▪▪▪▪▪□□□□□□□□□□▪▪□□□□▪▪▪▪▪▪▪	677777600060771	1 (0,24)	33,33	T	Unique
3038	▪▪□□□▪▪▪▪▪▪▪▪▪▪▪▪▪▪▪▪▪▪▪▪▪▪▪▪▪▪▪□□□□▪▪□□□□▪	617777777760601	1 (0,24)	33,33	T	Unique
3091	▪▪▪▪▪▪▪▪▪▪▪▪▪▪▪▪▪▪▪▪▪▪▪▪▪▪▪▪▪▪▪□□□□□□□□□▪▪▪	777777777740031	1 (0,24)	14,29	T	Unique
3139	▪□▪▪▪▪▪▪▪▪▪▪□▪▪▪▪▪▪▪▪▪▪▪▪▪▪▪▪▪▪▪□□□□▪▪▪▪▪▪▪	577737777760771	1 (0,24)	20	T3	Unique
3168*	▪▪▪□▪▪▪▪▪▪▪▪▪▪▪▪▪▪▪▪▪▪▪▪▪▪▪▪▪▪□▪□□□□▪▪▪□▪▪▪	737777777720731	3 (0,73)	75	H3	Clustered
3169*	▪□□▪▪□□□□□□□▪▪□□□□□□□□□□□□□□□□□□□□□□▪▪▪▪▪▪▪	460060000000771	2 (0,49)	66,67	Unknown	Clustered
3170*	▪▪□▪▪▪▪▪□▪▪▪▪▪▪□▪▪▪▪▪▪▪▪▪▪□▪▪▪▪▪▪▪▪▪▪▪□□□□□	676773776777600	3 (0,73)	75	BOV_1	Clustered
3171*	▪▪▪▪▪▪▪▪▪▪▪▪▪▪▪▪▪▪▪□□□□▪▪▪▪▪▪▪□▪□□□□▪▪▪▪▪▪▪	777777417720771	1 (0,24)	50	H3	Unique
3173*	▪▪▪▪▪▪□□□□□□□□□□□□▪▪▪▪▪▪▪▪▪□▪▪▪▪□□□□▪▪▪▪▪▪▪	770000777360771	2 (0,49)	66,67	T1-RUS2	Clustered
3175*	▪▪▪▪▪▪▪▪□□□□□▪□□□□▪▪▪▪▪▪▪▪▪▪▪▪▪▪□□□□▪▪▪▪▪▪▪	776020777760771	1 (0,24)	50	Unknown	Unique
3176*	▪▪▪▪▪▪▪▪▪▪□□□▪▪▪▪▪▪▪▪▪▪▪▪▪▪▪▪▪□▪□□□□▪▪▪▪▪▪▪	777437777720771	1 (0,24)	50	H3	Unique
3177*	▪▪▪▪▪▪□□□□□□□□□□□□□▪□□□□▪▪▪▪▪▪▪▪□□□□▪▪▪▪▪▪▪	770000207760771	1 (0,24)	50	Unknown	Unique
3179*	▪▪▪▪□▪▪▪▪▪▪▪▪▪▪▪▪▪▪▪▪▪▪▪▪▪▪▪▪▪□▪□□□□▪▪▪□▪▪▪	757777777720731	2 (0,49)	50	H3	Clustered
3180*	▪□□▪▪▪□□□□□□□□□□□□▪▪▪▪▪▪▪▪▪▪▪▪▪▪□□□□▪▪▪▪▪▪▪	470000777760771	2 (0,49)	66,67	T1-RUS2	Clustered
3181*	▪▪▪▪▪▪▪▪▪▪▪▪▪▪▪▪▪▪▪▪▪▪▪▪▪▪▪▪□▪□▪□□□□▪▪▪□▪▪▪	777777777520731	1 (0,24)	50	H3	Unique
3182*	▪▪▪▪▪▪▪▪▪▪▪▪▪▪▪▪▪□▪▪▪▪▪▪▪▪▪▪▪▪▪▪□□□□□□▪▪▪▪▪	777776777760171	1 (0,24)	50	X1	Unique
3183*	□□□□□□□□□□□□□□□□□□□□□▪▪▪▪▪▪▪▪▪▪▪□□□□▪▪▪▪▪▪▪	000000077760771	1 (0,24)	33,33	Unknown	Unique
3184*	▪▪□▪▪▪▪▪□▪▪▪▪▪▪□▪▪▪▪▪▪□□▪▪▪▪▪▪▪▪▪▪▪▪▪▪□□□□□	676773747777600	1 (0,24)	50	BOV_1	Unique
3185*	▪▪▪▪▪□□□▪▪▪▪▪▪▪▪▪▪▪▪▪▪▪▪▪▪▪▪▪▪□▪□□□□▪▪▪▪▪▪▪	761777777720771	2 (0,49)	100	H3	Clustered
3186*	▪▪▪▪▪▪▪▪▪▪▪▪▪▪▪▪▪▪▪▪▪▪▪□▪□□▪▪▪▪▪□□□□▪▪▪▪▪▪▪	777777764760771	1 (0,24)	50	T	Unique
3187*	▪□□▪▪□□□□□□□▪▪□□□□□□□□□□□□□□□□□□□□□□□▪▪▪▪▪▪	460060000000371	1 (0,24)	50	Unknown	Unique
3188*	▪▪▪□□□□□□□□□□□□□□□□□□□□□□□□□□□□□□□□□▪▪▪▪▪▪▪	700000000000771	1 (0,24)	50	Unknown	Unique
3189*	▪▪▪▪▪▪▪▪▪▪▪▪▪▪▪▪▪▪▪▪▪▪▪□▪□▪▪▪▪□▪□□□□▪▪▪▪▪▪▪	777777765720771	1 (0,24)	50	H3	Unique
3190*	▪▪▪▪▪▪▪▪▪▪▪▪▪▪▪▪▪▪▪▪▪▪□□▪▪▪▪▪▪▪▪□□□□▪□□□▪▪▪	777777747760431	1 (0,24)	50	T	Unique
3191*	▪□□□□□□□□□□□▪▪□□□□□□□□□□□□□□□□□□□□□□▪▪▪▪▪▪▪	400060000000771	1 (0,24)	50	Unknown	Unique
3199*	▪▪▪▪▪▪□□▪▪▪▪▪▪▪▪▪▪▪▪▪▪▪▪▪▪▪▪▪▪▪▪□□□□▪▪▪□▪▪▪	771777777760731	1 (0,24)	33,33	T2	Unique
3276*	▪▪▪▪▪▪▪▪▪▪▪▪▪▪▪▪▪▪▪▪▪▪▪▪□□□□□□□□□□□□□□▪▪▪▪▪	777777770000171	1 (0,24)	33,33	Unknown	Unique

A total of 104 SITs containing 332 isolates matched a preexisting shared type in the SITVIT2 database, whereas 23 SITs (n = 32 isolates) were newly-created either within the present study or after a match with an orphan in the database.

* SIT followed by an asterisk indicates “newly created shared-type” (n = 24 containing 34 isolates) due to two or more strains belonging to an identical new pattern within this study or after a match with an orphan in the database. SIT designations followed by number of strains: 3168* this study n = 3, PER n = 1; 3169* this study n = 2, USA n = 1; 3170* this study n = 3, SWE n = 1; 3171* this study n = 1, BRA n = 1; 3172* this study n = 1, VEN n = 1; 3173* this study n = 2, POL n = 1; 3174* this study n = 2; 3175 this study n = 1, USA n = 1; 3176* this study n = 1, ZAF n = 1; 3177* this study n = 1, GLP n = 1; 3178* this study n = 1, ALB n = 1; 3179* this study n = 2, FXX n = 2; 3180* this study n = 2, USA n = 1; 3181* this study n = 1, USA n = 1; 3182* this study n = 1, AUS n = 1; 3183* this study n = 1, FXX n = 1, ALB n = 1; 3184* this study n = 1, SWE n = 1; 3185* this study n = 2; 3186* this study n = 1, SWE n = 1; 3187* this study n = 1, SWE n = 1; 3188* this study n = 1, BEL n = 1; 3189* this study n = 1, DEU n = 1; 3190* this study n = 1, DEU n = 1; 3191* this study n = 1, SWE n = 1; 3199* this study n = 1, CHN n = 1, JPN n = 1; 3276* this study n = 1, COL n = 1, ITA n = 1.

** Spoligotype family designations according to SITVIT2 using revised SpolDB4 rules; “Unknown” designates patterns with signatures that do not belong to any of the major clades described in the database.

*** Clustered strains correspond to a similar spoligotype pattern shared by two or more strains “within this study”; as opposed to unique strains harboring a spoligotype pattern that does not match with another strain from this study. Unique strains matching a preexisting pattern in the SITVIT2 database are classified as SITs, whereas in case of no match, they are designated as “orphan”.

**Table 2:** Orphan strains (n = 45) and corresponding spoligotyping families/subfamilies found among a total of 409 *M. tuberculosis* complex strains isolated from Swedish patients born before 1945.

Isolate	Sex	Age	Spoligotype Description	Octal code	Spoligotypefamily
BTB09-066	F	92	▪▪□▪▪▪▪▪□▪▪▪▪▪□□□▪□□□□▪▪▪▪▪▪▪▪▪▪▪▪▪▪▪▪□□□□□	676761037777600	BOV_LIKE
BTB99-387	M	83	▪▪□▪▪▪▪▪□▪▪▪▪▪▪□□▪▪▪▪▪▪▪▪▪▪▪▪▪▪▪▪▪▪▪▪▪□□□□□	676771777777600	BOV-1
BTB07-032	M	87	▪▪▪▪▪▪▪▪▪▪□□□□□□□□□□□□□□▪□□□□□□▪□□□□▪▪▪▪▪▪▪	777400004020771	H1
BTB01-233	F	78	▪▪▪▪▪▪▪▪▪▪□□□□□□□□□□□□□□▪▪▪▪▪▪□▪□□□□▪▪▪▪▪▪▪	777400007720771	H3
BTB01-359	M	82	▪▪▪▪▪▪▪▪▪▪▪▪▪▪▪▪▪▪▪▪▪▪▪▪▪▪▪▪▪▪□▪□□□□▪▪□□□□□	777777777720600	H3
BTB05-050	M	70	▪▪▪▪▪▪▪▪▪▪▪▪▪▪▪▪▪▪▪▪▪▪▪▪▪▪▪▪▪▪□▪□□□□▪□▪□▪▪▪	777777777720531	H3
BTB05-143	M	63	▪▪▪▪▪▪▪▪▪▪▪▪□□□□□▪□▪▪▪▪▪▪▪▪▪▪▪□▪□□□□▪▪▪▪▪▪▪	777701377720771	H3
BTB05-149	F	63	▪▪▪▪▪▪▪▪▪▪▪▪▪▪▪▪▪▪□▪▪▪▪▪▪▪▪□□▪□▪□□□□▪▪▪▪▪▪▪	777777377120771	H3
BTB05-507	M	78	□□□□▪▪▪▪▪▪▪□▪▪▪▪□□▪▪▪▪▪▪▪▪▪▪▪□□▪□□□□▪▪▪▪▪▪▪	037674777620771	H3
BTB06-380	M	88	▪▪▪▪▪▪▪▪▪▪▪□□□□□□□□□□□□□□□□□□□□▪□□□□▪▪▪▪▪▪▪	777600000020771	H3
BTB06-563	M	79	▪▪▪▪▪▪▪▪▪□□□□□□□□□□□□□□□□□□□□□□▪□□□□▪□▪▪▪▪▪	777000000020571	H3
BTB06-654	F	83	▪▪▪▪▪▪▪▪□□□□▪▪▪▪▪▪▪▪▪▪□□▪▪▪▪▪▪□▪□□□□▪▪□▪▪▪▪	776077747720671	H3
BTB07-067	F	64	▪▪□□□□□□□□□□□□□□□□□▪▪▪▪▪▪▪▪▪▪▪□▪□□□□▪▪▪▪□▪▪	600000377720751	H3
BTB07-320	F	82	▪▪▪▪▪▪▪▪▪▪▪▪□▪▪▪▪▪▪□▪▪▪▪▪▪▪▪□□□▪□□□□▪▪▪▪▪▪▪	777737577420771	H3
BTB09-374	F	79	▪▪□□□□□□□□▪▪▪▪▪▪▪▪▪▪▪▪▪▪▪▪▪▪▪▪□▪□□□□▪▪▪▪▪▪▪	600377777720771	H3
BTB09-435	F	98	▪▪▪▪▪▪▪▪▪▪□□□□□□□□□□□□□□▪□□□□▪□▪□□□□▪▪▪▪▪▪▪	777400004120771	H3
BTB99-379	M	55	▪▪▪▪▪▪▪▪▪▪▪▪□▪▪▪▪▪▪▪▪▪□□▪▪▪▪▪▪□▪□□□□▪▪▪▪▪▪▪	777737747720771	H3
S96-202	M	82	▪□▪□□□□□□▪▪▪▪▪▪□□▪▪▪▪▪□□▪□□□□□□▪□□□□▪▪▪□▪▪▪	500771744020731	H3
BTB00-127	M	89	□□□□□□□□□□□□□□□□□□□□□□□□▪▪▪▪□□□▪□□□□▪▪▪□▪▪▪	000000007420731	H4
BTB05-475	M	85	▪▪□▪▪▪▪▪▪▪▪▪▪▪▪▪▪▪▪▪□□□□▪▪▪▪▪▪▪▪□□□□▪▪▪□▪▪□	677777607760730	LAM
BTB04-450	M	83	▪▪▪▪▪▪▪▪▪▪▪▪▪▪▪□□▪□▪▪▪□▪□□□□□□▪▪□□□□▪▪▪▪▪▪▪	777771350060771	T
BTB05-005	F	61	▪▪▪▪▪▪□□□□□□□□□□□□▪▪□▪▪▪▪▪▪▪▪▪▪▪□□□□▪▪▪□▪▪▪	770000677760731	T
BTB98-539	M	77	▪▪▪▪▪▪□□□□□□□□□□□□▪▪□□□□▪▪▪▪▪▪▪▪□□□□▪▪▪□▪▪▪	770000607760731	T
BTB00-056	F	76	▪▪▪▪▪▪▪▪▪▪▪▪▪▪▪▪▪▪▪▪▪▪□□▪▪▪▪▪▪▪▪□□□□▪□□□□▪▪	777777747760411	T1
BTB00-059	M	83	▪▪▪▪▪▪▪▪▪▪▪▪▪▪▪▪□▪▪▪▪▪□□▪▪▪▪▪▪▪▪□□□□▪□□▪▪▪▪	777775747760471	T1
BTB01-392	M	72	▪▪▪▪▪▪▪▪▪▪▪□□□□□□□□□□□□□□□▪▪▪▪▪▪□□□□▪▪▪□□▪▪	777600001760711	T1
BTB05-487	M	87	□□□□□□□□□□□□□□□□□□□□▪▪▪▪▪▪□▪▪▪▪▪□□□□▪▪▪▪▪▪▪	000000176760771	T1
BTB07-129	F	93	▪▪▪□▪▪□▪▪▪▪▪▪▪▪▪▪▪▪▪▪▪▪▪▪▪▪▪▪▪▪▪□□□□▪▪▪□▪▪▪	733777777760731	T1
BTB07-182	M	84	▪▪□▪▪▪▪▪▪▪▪▪▪▪▪▪▪▪▪▪▪▪▪▪▪▪▪□▪▪▪▪□□□□□▪▪▪▪▪▪	677777777360371	T1
BTB07-232	M	83	▪▪▪▪▪▪▪▪▪▪▪▪▪▪▪▪▪▪▪▪▪▪□▪▪▪▪▪□□▪▪□□□□▪▪▪▪▪▪▪	777777757460771	T1
BTB08-128	F	75	▪▪▪▪▪▪□▪▪▪▪▪▪▪▪▪▪▪□□□□▪▪▪▪▪▪▪▪▪▪□□□□▪▪▪▪▪▪▪	773777037760771	T1
BTB09-443	F	93	□□□□□□□□□□□▪▪▪▪▪▪▪▪▪▪□□▪▪▪▪▪▪▪▪▪□□□□▪▪▪▪▪▪▪	000177717760771	T1
BTB09-515	M	77	▪▪▪▪▪▪▪▪▪▪▪▪▪▪□▪▪▪▪▪▪▪□▪□□□□□□▪▪□□□□▪▪▪▪▪▪▪	777767750060771	T1
BTB98-359	F	80	▪▪▪▪▪▪▪▪▪▪▪▪▪▪▪▪▪▪▪▪□□□□□▪□□□□▪▪□□□□□▪▪▪▪▪▪	777777602060371	T1
BTB98-532	F	85	□▪▪▪▪▪▪▪▪▪▪□▪▪▪▪▪▪▪▪▪▪▪▪▪▪▪▪▪▪▪▪□□□□▪▪▪▪▪▪▪	377677777760771	T1
BTB98-547	M	60	▪▪□□□□□▪▪▪▪▪▪▪▪▪▪▪▪▪▪▪▪▪▪▪▪▪▪▪▪▪□□□□▪▪▪▪▪▪▪	603777777760771	T1
BTB99-138	F	80	▪▪▪□▪▪▪▪▪▪▪▪▪▪▪▪▪▪▪▪▪▪▪▪▪▪▪▪▪▪▪▪□□□□▪▪▪□▪□□	737777777760720	T1
BTB99-476	F	85	▪▪▪▪▪▪▪▪▪▪▪▪▪▪▪▪▪▪▪▪▪▪▪▪▪▪▪□▪▪▪▪□□□□□▪▪▪▪▪▪	777777777360371	T1
S96-295	F	64	▪▪▪▪▪▪□□□□□□□□□□□□▪▪□▪□□□▪▪▪▪▪▪▪□□□□▪▪▪▪▪▪▪	770000643760771	T1-RUS2
BTB08-174	F	89	▪▪▪▪□▪▪▪▪▪▪▪▪▪▪▪▪▪▪▪▪▪▪□▪▪▪▪▪▪▪▪□□□□▪▪▪□▪▪▪	757777767760731	T2
BTB07-190	M	77	▪▪▪□□□▪▪▪▪▪▪▪▪▪▪▪▪□▪▪▪□□▪□□▪□▪▪▪□□□□▪□□▪▪▪▪	707777344560471	T4-CEU1
BTB99-125	F	61	▪▪▪▪□▪▪▪▪▪▪▪▪▪▪▪▪▪▪▪▪▪□▪▪▪▪▪▪▪▪▪□□□□▪▪▪□□▪▪	757777757760711	T5
BTB04-418	F	89	▪▪▪▪▪▪▪▪▪▪▪▪▪▪▪▪▪▪▪▪▪▪□□▪▪▪▪▪▪□□□□□□▪▪▪▪▪▪▪	777777747700771	Unknown
BTB09-069	F	80	▪▪▪▪□□□□□□□□□□□□□□□□□□□□□□□□□□□□□□□□□□□□▪▪▪	740000000000031	Unknown
BTB98-543	M	69	▪▪▪▪▪▪▪▪□□▪▪▪▪▪▪▪▪▪▪▪▪▪▪▪▪▪▪▪▪▪□□□□□□□□□□□□	776377777740000	Unknown

* Spoligotype family designations for orphan patterns were done manually as Expert-based interpretations using revised SpolDB4 rules.

The 168 patients with H family isolates were born between 1908–1945 (median 1924), with a mean age of 77.8 years at diagnosis (range 52–98), the 154 patients with T family isolates were born between 1910–1945 (median 1923), with a mean age of 78.5 years at diagnosis (range 53–97), and the 32 patients with LAM family isolates were born between 1913–1943 (median 1923), with a mean age of 78.9 years at diagnosis (range 63–93). The three CAS1-Delhi strains were isolated from patients born in 1921, 1938, and 1943 with a mean age of 67.7 years (57, 71, and 75 years). The four patients with EAI isolates were born later than the patients with H, T and LAM isolates. They were all except one born in the 1940s: three EAI2-Manilla isolates from patients born 1911, 1943 and 1943 and one EAI5 isolate from a patient born 1943. The patients were also younger (mean age 68.0) than average at diagnosis (57, 62, 66 and 87 years). The three patients with Beijing isolates were all diagnosed between 2007 and 2008, i.e. in the later part of the study, at a mean age of 80.7 years. We tested the hypothesis that the 363 “modern” isolates of the H, T, LAM, X and S families differed in patient characteristics compared to the 24 “ancient” isolates split into two groups [group one (n = 10): Beijing, EAI and CAS and group two (n = 14): *M. bovis/M. bovis* like and Manu] with regard to the age of the patients at diagnosis and date of birth. Patients with “modern” isolates of the H, T, LAM, X and S families were significantly older at diagnosis and were born significantly earlier than patients with “ancient” isolates.

**Figure 1:**
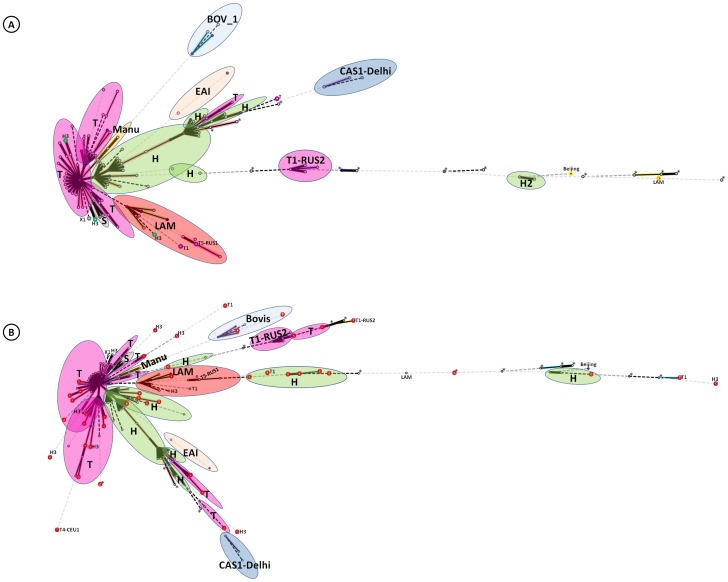
A minimum spanning tree (MST) illustrating possible evolutionary relationships between the Swedish spoligotypes obtained for *M. tuberculosis* complex strains causing the TB epidemic a century ago. (**A**) **SITs alone** (**B**) **all patterns (including the orphan patterns) pooled together.** The tree connects each genotype based on degree of changes required to go from one allele to another. The structure of the tree is represented by branches (continuous vs. dotted lines) and circles representing each individual pattern. Note that the length of the branches represents the distance between patterns while the complexity of the lines (continuous, black dotted and gray dotted) denotes the number of allele/spacer changes between two patterns: solid lines, 1 or 2 change (thicker ones indicate a single change, while the thinner ones indicate 2 changes); dotted lines, three or more changes (black dotted for 3, and grey dotted for 4 or more changes). The color of the circles is proportional to the number of clinical isolates in our study, illustrating unique isolates (sky blue) versus clustered isolates (blue, 2–5 strains; dark blue, 6–9 strains; bordeaux, 10–19 strains; red, 20 and more). Abbreviation: PGG, Principal Genetic Group. Note that orphan patterns in Fig. 1B are circled in red while patterns marked by an asterisk (*) indicate an strain with an unknown signature (unclassified).

For phylogenetical analyses, we drew two separate MSTs (Figure 1) to summarize the possible evolutionary relationships between all the genotypes obtained. Figure 1A is based only on SITs, while Figure 1B shows combined data for both SITs and orphan patterns pooled together. The SIT-based MST (Figure 1A) shows a tree split into distinct families: the top section displays the ancient PGG1 strains (EAI, Bovis, Manu and CAS1-Delhi families) whereas the bottom shows the evolutionary modern PGG2/3 strains belonging to the H, T and LAM families. As summarized in Table 2, it should be noticed that only 2/47 orphan strains were PGG1 (1 BOV-1, and 1 BOV_LIKE), the rest being evolutionary modern PGG2/3. The spoligoforests generated (see Figures S1 and S2, and legends for detailed comments) highlighted the predominance of PGG2/3 group which are well represented (as large, visible nodes). The rare ancestral PGG1 strains (belonging to the EAI, Manu, and CAS families) were mostly located in the top layer of the hierarchical layout as isolated strains without interconnections with others (Figure S1).

Interestingly, the MST shown after combining orphans with SITs in Figure 1B is overlapping with the tree shown in Figure 1A in the sense that the two ancestral PGG1 orphans were grouped together with their own family (*M. bovis*), while evolutionary modern PGG2/3 (H, T, LAM) SITs grouped with their own orphans. Almost all of the evolutionary-recent orphan strains appeared at distant (terminal) positions within their respective genotypic families on the tree and not as the central nodes that were essentially defined by spoligotype family prototypes such as SIT50 for H3 and SIT53 for T1 subfamilies. This observation suggests that most of the orphan strains that existed in Sweden a century ago, when TB was highly endemic, probably underwent extinction because they evolved as terminal members within these families that were unfit to proliferate. Indeed, in the Fruchterman-Reingold spoligoforest tree, many PGG2/3 orphans are not linked to other nodes (Figure S2).

### IS*6110* RFLP

**Figure 2:**
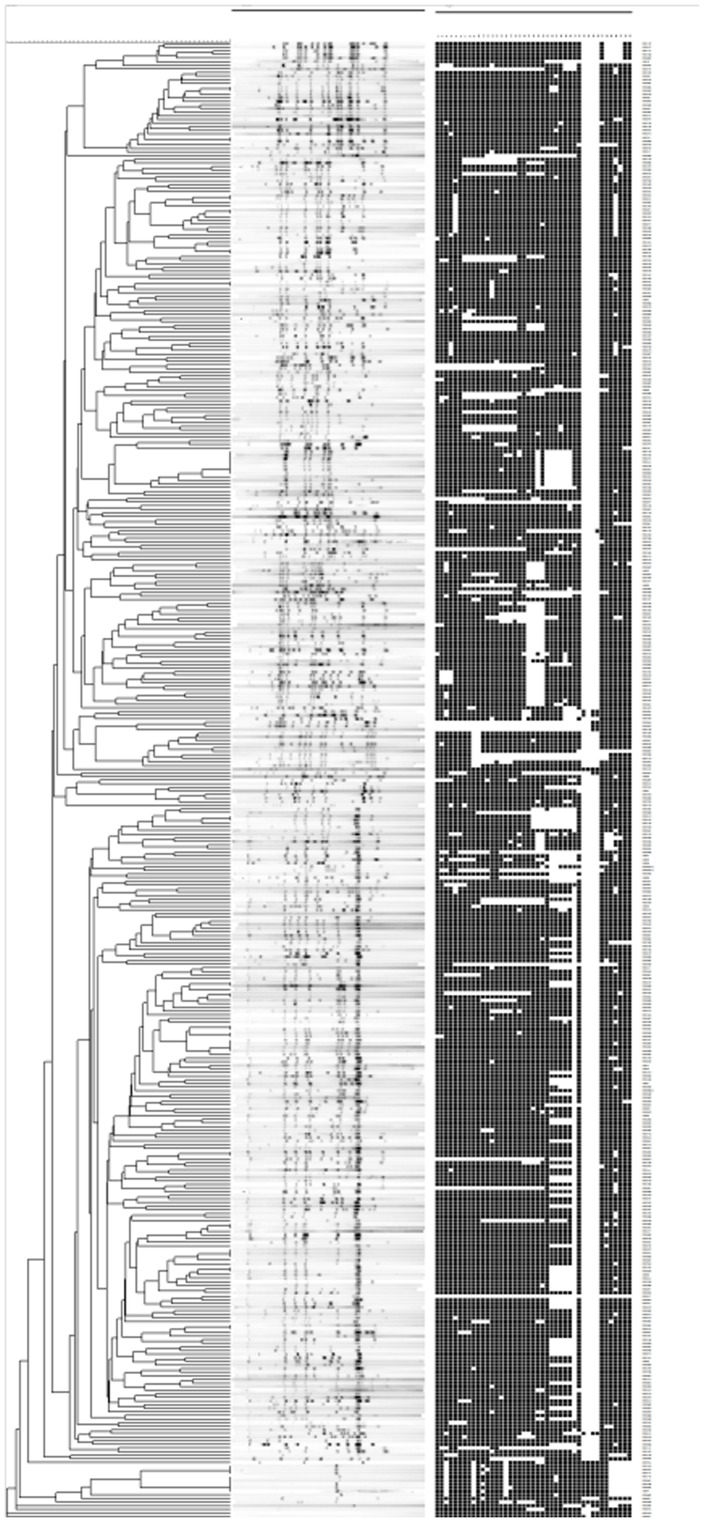
IS*6110* Restriction Fragment Length Polymorphism and spoligotyping dendrogram of 409 *M. tuberculosis* complex strains from 409 patients born in Sweden before 1945.

Out of 409 isolates, 375 different RFLP patterns were obtained, of which 53 (13.0%) were clustered in 19 RFLP clusters comprising 2–9 isolates per cluster (Figure 2). Most clusters were small and the majority comprised only two individuals (n = 15). The remaining 356 (87.0%) RFLP patterns were unique i.e. the strains did not cluster with other patient strains in the SMI database. A clustering comparison was also made with isolates from patients born in Sweden in 1985 or later. It was found that present day isolates cluster much more frequently. Among 58 isolates from these young Swedes, 16 (27.6%) belonged to a cluster compared to 53/409 (13.0%) among the elderly Swedes (chi^2^-test p-value 0.0033). All clusters (n = 5) among the young Swedes also contained a foreign born patient with an isolation date prior to the young Swedish born patients.

## Discussion

In this study of *M. tuberculosis* complex isolates from patients born in Sweden before 1945 the majority of the isolates should represent the epidemic strains circulating at the time of the down slope of the epidemic that took place in Sweden the last centuries. The shape of the epidemic curve for TB is the same as that for any other infectious disease, if one adjusts the time scale to allow for the roughly 300-year duration of a TB epidemic [22]. Rates of mortality, morbidity and latent infections increase rapidly, peak successively, and decline at a prolonged, exponentially decelerated pace [22]. In Europe the epidemic started over three centuries ago, and the TB morbidity and mortality began to fall well before introduction of BCG vaccine and effective treatment. It began in the late 16^th^ century in England, where it peaked around the 1780s [10]. From England it spread rapidly eastwards, to developing cities on the continent, where the curves peaked in the first decades of the 19^th^ century. In Scandinavia, the peaks in mortality were reached in the last decades of the 19^th^ century [10], and the incidence declined to 147 per 100.000 population in 1950 and to 1.4 in 2009 in the Swedish-born population. A similar shape of the epidemic curve can be observed in the neighbouring Scandinavian countries. The present TB situation in Sweden is largely influenced by migration as only 11% of the patients diagnosed in 2011 were born in Sweden. Approximately 40% of all isolates typed in 2011 belonged to a cluster [6]. The low rate of isolates clustered by RFLP in the elderly population studied here supports the concept that most isolates represent reactivation cases, without active TB transmission, and thus represent the past epidemic. Indeed, a significant difference in clustering was found when this elderly Swedish population was compared to a young Swedish population.

We found a highly homogenous bacterial population with a domination of the H (41.1%), T (37.7%), and LAM (7.8%) families, which belong to the modern Euro-American group of strains and includes all the spoligotype families predominating in the Western world, such as H, LAM, the ill-defined T group, X and S. Only 24 isolates did not belong to modern families. This high prevalence of modern H, T and LAM strains is similar to the prevalence among isolates from patients born before 1950 in Norway, where a total of 40% of 213 isolates belonged to the T family and 35% to the H family [23]. As the Norwegian study, our study only included isolates displaying RFLP patterns not previously present in our database indicating an unlikely recent transmission. Both studies demonstrated that the isolates were of a highly homogenous population (T and H family) with low rate of diversity. The two major spoligotypes in our study were SIT50 of the H3 subfamily, and SIT53 of the T1 subfamily. Although they have been designated to two different subfamilies, they only differ in one spacer. In addition to the lack of spacers 33–36 in both types SIT50 also lacks spacer 31. SIT53 is the prototype pattern of the T family and is widely spread in the world [11]. The T family defines a polyphyletic group of strains belonging to the Euro-American superlineage. It does not represent a lineage in a strict evolutionary sense since it was defined by default [11]. Although, some subfamilies belonging to the T group have a known phylogeographical specificity, i.e. T-Tuscany, T1-RUS2 (Russia), T2-Uganda, T3-ETH (Ethiopia), T3-OSA (Osaka), T4-CEU1 (Central Europe), T5-Madrid2, the remaining T1 to T5 spoligo-prototypes are not monophyletic [24]. In a molecular analysis of *M. tuberculosis* DNA from a family of 18th century Hungarians two spoligotypes were identified, corresponding to SIT50 and SIT53 [25]. As in our study SIT53 is found in elderly patients in other parts of the world: in a study from Venezuela, patients with SIT53 had a significantly higher mean age compared to all other patients [26], and in Mexico SIT53 was significantly more prevalent in elderly patients, especially in females [27]. Polymorphism in the direct repeat region in clinical isolates appears to be the result of successive deletions of single discrete direct variant repeats (DVRs) or of multiple contiguous DVRs from a primordial direct repeat region [28]. Thus the lack of spacer block 33–36 in SIT53 is a very large signature that defines almost all evolutionary modern PGG2/3 *M. tuberculosis* strains. It also corresponds to the large sequence polymorphism-based broader “Euro-American superlineage”. Thus it can be seen as the baseline structure among evolutionary-modern *M. tuberculosis* complex strains (defined by prototype SIT53). From it might have evolved most of other “modern” strains by loss of spacers, including the H family strains. The H baseline therefore is defined by prototype SIT50 of the H3 subfamily (which has a characteristic absence of spacer 31). In addition to the T1 subfamily prototype, SIT53, two more T clade SITs (SIT37 and SIT153) were among the seven predominant SITs. It can be speculated that they separately derived from the prototype SIT53 by loss of spacer 13 (SIT37), or the loss of spacers 5 and 40 (SIT153). However, to know whether such differences are caused by a single or multiple event(s), one would need complementary investigations.

We only found 24 ancestral PGG1 *M. tuberculosis* strains, including isolates of the EAI2-Manila and CAS families. EAI2-Manila isolates are more commonly found in Asian countries, such as Indonesia or the Philippines, where they account for high percentages of the *M. tuberculosis* isolates. Ancestral PGG1 strains are usually linked to migrants in todaýs Sweden [4], [29], indicating they are more recently introduced in Sweden. The majority of the isolates collected in Sweden present in the database at SMI are from foreign born patients. Only three isolates were of the recently emerging Beijing genotype. The majority of patients with drug resistant Beijing strains in Sweden are foreign born [30]. The three patients with Beijing isolates were all diagnosed between 2007 and 2008, i.e. in the later part of the study, at a mean age of 80.7 years. Since the Beijing family is recently introduced in Sweden [30], it is possible that these cases are recent infections and not reactivation of earlier infections.

In the 1930s and 1940s bovine TB was considered to be a significant zoonosis in Europe, including Sweden, and *M. bovis* was thought to be responsible for more than 50% of cervical lymphadenitis cases in children [31] through milk and dairy products from infected herds. In 1958, Sweden was declared free of bovine TB after an extensive national eradication campaign, by the long term application of a test-and-slaughter policy that removed infected cattle [32]. Thus, the 11 *M. bovis* isolates are all from patients born before 1958, indeed the youngest persons with *M. bovis* infection were born in 1911. The four SIT691 isolates have previously been shown to contain the RDEU1 deletion [33] which is specific for the clonal complex European 1 (Eu1), the dominant *M. bovis* complex isolated from cattle in the Republic of Ireland and the UK.

The fact that patients with isolates of the H, T and LAM families were significantly older at diagnosis, and were born significantly earlier than patients with “ancient” isolates further supports the hypothesis that the closely knit H, T, and LAM isolates represent the old TB epidemic in Sweden, and probably the whole of Scandinavia. In the study from Venezuela, patients with SIT53 were not only older but were more commonly smear negative. The authors draw the conclusion that SIT53 strains may be less virulent and associated with reactivation of past infections in older patients. These associations provoke a number of questions. If SIT53 were really less virulent, why is it still the sixth most common spoligotype in SITVIT2, causing 4% of cases? Could it have been a very common strain in the past, that is now more apt at latency and reactivation than person-to-person transmission, and will its prevalence decrease over time? One could speculate that SIT53 represents a progenitor of the strains causing the epidemic in Sweden and Norway, where mutations causing attenuation of the outbreak strains are illustrated by the further loss of spacers in e.g. SIT50, and SIT153.

Although spoligotyping is known to show marked homoplasy [34], we find the fact interesting that evolutionary recent orphan strains of the PGG2/3 grouping essentially appeared at terminal positions within their respective genotypic families on the MST. The fact that none constituted the central nodes within their own major genotypic families nor appeared to play an important role in the interconnection of prevailing families, suggests why they are probably not at the heart of the *M. tuberculosis* biodiversity observed in Sweden. It is possible that these orphan strains correspond to strains that were simply not represented well enough in the Swedish TB epidemic to have continued on infecting people. However, we believe that most of these orphan strains probably underwent extinction because they were the most vulnerable and unfit strains to be able to continue infecting new hosts during the epidemic peak of the disease a century ago. A detailed genetic analysis might discover a link to a lack of fitness of these strains. In conclusion the study of genetic variability within natural populations of pathogens may provide insight into bacterial evolution and pathogenesis. The characteristics of the bacterial population studied here are those of an old outbreak under extinction, with the superimposition of new characteristics due to cases of importation and recent transmission.

## Supporting Information

Figure S1 **Hierarchical layout based spoligoforest tree showing only the shared-type SIT patterns.** The spoligoforest trees are drawn using http://www.emi.unsw.edu.au/spolTools In this figure each spoligotype pattern is represented by a circle with the area proportional to the number of isolates with that pattern (in the circles SIT numbers are shown followed by the number of isolates with that pattern). Note that contrary to the Minimum spanning trees, the Spoligoforest trees are directed, and only evolve by loss of spacers. Comments on Figure S1: The Hierarchical layout based tree shown in Figure S1 represents hierarchically the changes between *Mycobacterium tuberculosis* complex strains; the more the strains evolve (lose spacers), the more they are present in the down layouts (bottom of the figure, like tree leaves). The patterns which have not lost many alleles/spacers are located in the upper layouts. However, in case of too many changes between two spoligotype patterns, there are no links/edges linking them (like some of the orphan patterns shown as the smallest circles on the top). In this method, the authors used a heuristic method that selects a single inbound edge with a maximum weight using a Zipf model; solid black lines link patterns which have a maximum weight of distance (very similar: loss of one spacer). Dashed line represents a link of weight comprised between 0.5 and 1. And dotted line represents a link of weight less than 0.5. In our study sample, one can notice the predominance of evolutionary recent *M. tuberculosis* spoligotype families (Haarlem, ill-defined T, and LAM): SIT50/H3 (n = 56), SIT53/T1 (n = 43), SIT47/H1) (n = 33); followed by SIT42/LAM9 (n = 14). Regarding some interesting evolutionary conclusions, one may notice; (i) on the top left of the figure, that SIT26/CAS1-Delhi leads to SIT1/Beijing as a second generation descendant (via SIT485/CAS1-Delhi); (ii) on the top center of the figure, patterns belonging to Manu family may evolve to highly represented (and ubiquitous) SIT53/T1 spoligotype family.
(PDF)

Figure S2 **Fruchterman Reingold algorithm based spoligoforest tree of all spoligotype patterns including orphans.** The spoligoforest trees are drawn using http://www.emi.unsw.edu.au/spolTools In this figure each spoligotype pattern is represented by a circle with the area proportional to the number of isolates with that pattern (in the circles SIT numbers are shown followed by the number of isolates with that pattern). Note that contrary to the Minimum spanning trees, the Spoligoforest trees are directed, and only evolve by loss of spacers. Comments on Figure S2: The Fruchterman Reingold algorithm based tree shown in Figure S2 essentially represents the same relationships as in the Hierarchical layout, but the nodes containing a huge number of strains are centered and more visible in the figure. Considering that the patterns evolve by loss of spacers, the proximity of the nodes represents similarity between them. Note that this figure was drawn without orphan patterns to better show the SIT numbers (in circles) followed by the number of strains for each pattern (shown in brackets). The interpretation of the links (solid black, dashed, or dotted) is the same as for Hierarchical layout.
(PDF)

Table S1 **Patients born in Sweden between the years 1908–1945.**
(DOCX)

Table S2 **Patients born in Sweden between the years 1985–2008.**
(DOCX)

Table S3 **Description of predominant SITs (patterns representing ≥2% strains in our study), and their worldwide distribution.**
(DOCX)

Table S4 **Description of rare PGG1 spoligotype patterns in this study (n = 15 patterns containing 24 isolates), and their worldwide distribution.**
(DOCX)
