# Supplementary figures and images for: Mycobacterium tuberculosis Strains Potentially Involved in the TB Epidemic in Sweden a Century Ago
Source: PLoS One. 2012 Oct 8;7(10):e46848. doi: 10.1371/journal.pone.0046848 (PMC3466202; doi:10.1371/journal.pone.0046848)

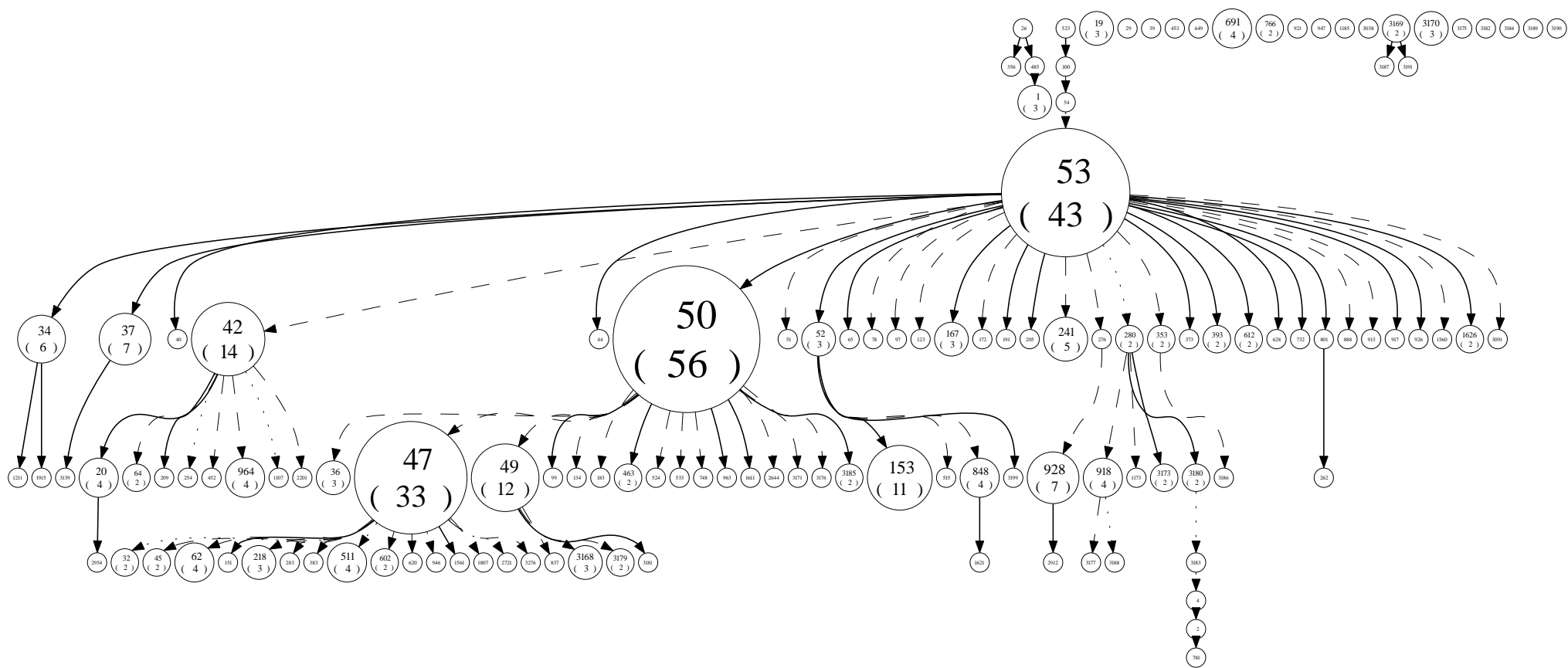

Supplement: Figure S1 — Hierarchical layout based spoligoforest tree showing only the shared-type SIT patterns. The spoligoforest trees are drawn using http://www.emi.unsw.edu.au/spolTools In this figure each spoligotype pattern is represented by a circle with the area proportional to the number of isolates with that pattern (in the circles SIT numbers are shown followed by the number of isolates with that pattern). Note that contrary to the Minimum spanning trees, the Spoligoforest trees are directed, and only evolve by loss of spacers. Comments on Figure S1: The Hierarchical layout based tree shown in Figure S1 represents hierarchically the changes between Mycobacterium tuberculosis complex strains; the more the strains evolve (lose spacers), the more they are present in the down layouts (bottom of the figure, like tree leaves). The patterns which have not lost many alleles/spacers are located in the upper layouts. However, in case of too many changes between two spoligotype patterns, there are no links/edges linking them (like some of the orphan patterns shown as the smallest circles on the top). In this method, the authors used a heuristic method that selects a single inbound edge with a maximum weight using a Zipf model; solid black lines link patterns which have a maximum weight of distance (very similar: loss of one spacer). Dashed line represents a link of weight comprised between 0.5 and 1. And dotted line represents a link of weight less than 0.5. In our study sample, one can notice the predominance of evolutionary recent M. tuberculosis spoligotype families (Haarlem, ill-defined T, and LAM): SIT50/H3 (n = 56), SIT53/T1 (n = 43), SIT47/H1) (n = 33); followed by SIT42/LAM9 (n = 14). Regarding some interesting evolutionary conclusions, one may notice; (i) on the top left of the figure, that SIT26/CAS1-Delhi leads to SIT1/Beijing as a second generation descendant (via SIT485/CAS1-Delhi); (ii) on the top center of the figure, patterns belonging to Manu family may evolve to highly rep [file pone.0046848.s001.pdf]

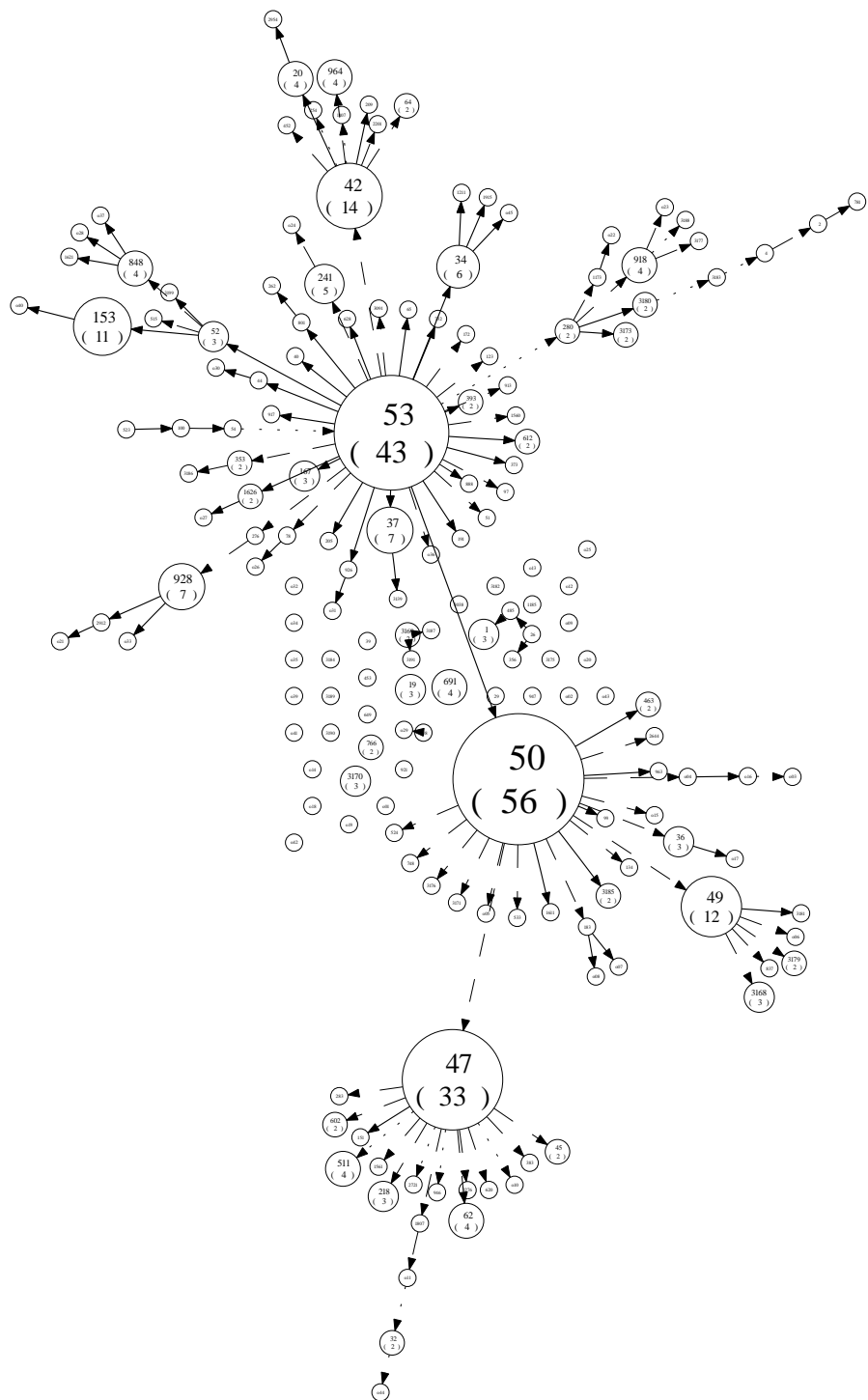

Supplement: Figure S2 — Fruchterman Reingold algorithm based spoligoforest tree of all spoligotype patterns including orphans. The spoligoforest trees are drawn using http://www.emi.unsw.edu.au/spolTools In this figure each spoligotype pattern is represented by a circle with the area proportional to the number of isolates with that pattern (in the circles SIT numbers are shown followed by the number of isolates with that pattern). Note that contrary to the Minimum spanning trees, the Spoligoforest trees are directed, and only evolve by loss of spacers. Comments on Figure S2: The Fruchterman Reingold algorithm based tree shown in Figure S2 essentially represents the same relationships as in the Hierarchical layout, but the nodes containing a huge number of strains are centered and more visible in the figure. Considering that the patterns evolve by loss of spacers, the proximity of the nodes represents similarity between them. Note that this figure was drawn without orphan patterns to better show the SIT numbers (in circles) followed by the number of strains for each pattern (shown in brackets). The interpretation of the links (solid black, dashed, or dotted) is the same as for Hierarchical layout. (PDF) [file pone.0046848.s002.pdf]
