# Supplementary material for: Mycobacterium tuberculosis Strains Potentially Involved in the TB Epidemic in Sweden a Century Ago
Source: PLoS One. 2012 Oct 8;7(10):e46848. doi: 10.1371/journal.pone.0046848 (PMC3466202; doi:10.1371/journal.pone.0046848)
Supplement: Table S1 — Patients born in Sweden between the years 1908–1945. (DOCX) [file pone.0046848.s003.docx]

**Table S1.** Patients born in Sweden between the years 1908-1945.

| **Born** | **Number of patients (%)** | **Mean (median) age at diagnosis** |
| --- | --- | --- |
| <1914 | 40 (9.8%) | 88 (86) |
| 1915-1919 | 80 (19.6%) | 85 (85) |
| 1920-1924 | 111 (27.1%) | 80 (81) |
| 1925-1929 | 70 (17.1%) | 77 (78) |
| 1930-1934 | 45 (11.0%) | 73 (74) |
| 1935-1939 | 30 (7.3%) | 67 (69) |
| 1940-1945 | 33 (8.1%) | 60 (61) |
