# Supplementary material for: Mycobacterium tuberculosis Strains Potentially Involved in the TB Epidemic in Sweden a Century Ago
Source: PLoS One. 2012 Oct 8;7(10):e46848. doi: 10.1371/journal.pone.0046848 (PMC3466202; doi:10.1371/journal.pone.0046848)
Supplement: Table S2 — Patients born in Sweden between the years 1985–2008. (DOCX) [file pone.0046848.s004.docx]

**Table S2.** Patients born in Sweden between the years 1985-2008.

| **Born** | **Number of patients (%)** | **Mean (median) age at diagnosis** |
| --- | --- | --- |
| 1985-1989 | 14 (24.1%) | 19 (20) |
| 1990-1994 | 22 (37.9%) | 13 (15) |
| 1995-1999 | 10 (17.2%) | 5 (5) |
| 2000-2004 | 5 (8.6%) | 3 (3) |
| 2005> | 7 (12.1%) | 1 (1) |
