# Supplementary material for: Mycobacterium tuberculosis Strains Potentially Involved in the TB Epidemic in Sweden a Century Ago
Source: PLoS One. 2012 Oct 8;7(10):e46848. doi: 10.1371/journal.pone.0046848 (PMC3466202; doi:10.1371/journal.pone.0046848)
Supplement: Table S3 — Description of predominant SITs (patterns representing ≥2% strains in our study), and their worldwide distribution. (DOCX) [file pone.0046848.s005.docx]

**Table S3.** Description of predominant SITs (patterns representing ≥2% strains in our study), and their worldwide distribution.

| **SIT (Clade) Octal Number** | **Number (%) in this study** | **% in study vs. database** | **Distribution in Regions with ≥ 3% of a given SITs *** | **Distribution in countries with ≥ 3% of a given SITs **** |
| --- | --- | --- | --- | --- |
| **Spoligotype Description** |  |  |  |  |
| 42 (LAM9)777777607760771 | 14 (3.42) | 0.44 | AMER-S 30.34, AMER-N 14.25, EURO-S 10.45, EURO-W 9.89, AFRI-N 8.95, EURO-N 3.9, AFRI-E 3.71, AFRI-S 3.31 | BRA 12.5, USA 12.38, COL 7.95, MAR 7.36, ITA 5.46, FXX 5.27, ESP 3.49, VEN 3.46, ZAF 3.31 |
| ■■■■■■■■■■■■■■■■■■■■□□□□■■■■■■■■□□□□■■■■■■■ |  |  |  |  |
| 47 (H1)777777774020771 | 33 (8.07) | 2.26 | EURO-W 21.78, AMER-N 18.36, EURO-S 14.45, AMER-S 12.12, EURO-E 7.06, EURO-N 6.78, AFRI-N 3.9, ASIA-W 3.7 | USA 16.3, ITA 8.84, AUT 8.63, BRA 7.53, FXX 7.12, CZE 4.04, ESP 3.84, SWE 3.63, MAR 3.01 |
| ■■■■■■■■■■■■■■■■■■■■■■■■■□□□□□□■□□□□■■■■■■■ |  |  |  |  |
| 49 (H3) 777777777720731 | 12 (2.93) | 6.98 | EURO-N 24.42, EURO-W 18.61, AMER-N 17.44, EURO-S 12.21, AMER-S 11.05, AFRI-M 4.65 | USA 15.12, FIN 13.37, FXX 12.21, SWE 11.05, ITA 6.98, PER 5.23, PRT 4.07, AUT 4.07 |
| ■■■■■■■■■■■■■■■■■■■■■■■■■■■■■■□■□□□□■■■□■■■ |  |  |  |  |
| 50 (H3) 777777777720771 | 56 (13.69) | 1.71 | AMER-N 18.91, EURO-W 18.12, AMER-S 17.63, EURO-S 11.91, EURO-E 5.48, EURO-N 4.42, AFRI-N 4.39, AFRI-S 4.17, CARI 3.59 | USA 18.09, BRA 7.28, FXX 7.1, AUT 6.27, ITA 5.6, ESP 5.6, PER 4.23, ZAF 4.17, CZE 3.78 |
| ■■■■■■■■■■■■■■■■■■■■■■■■■■■■■■□■□□□□■■■■■■■ |  |  |  |  |
| 53 (T1) 777777777760771 | 43 (10.51) | 0.72 | AMER-N 17.1, EURO-W 16.38, AMER-S 12.48, EURO-S 9.85, ASIA-W 7.13, EURO-N 5.58, AFRI-S 5.2, AFRI-E 4.72, ASIA-E 4.43, AFRI-N 3.68 | USA 13.82, FXX 8.25, ITA 5.58, BRA 5.37, ZAF 5.08, TUR 3.63, AUT 3.58, CHN 3.23 |
| ■■■■■■■■■■■■■■■■■■■■■■■■■■■■■■■■□□□□■■■■■■■ |  |  |  |  |
| 153 (T2) 757777777760731 | 11 (2.69) | 14.86 | EURO-S 28.38, EURO-N 24.32, AMER-S 13.51, AMER-N 9.46, ASIA-E 8.11, EURO-W 6.76, CARI 6.76 | ITA 28.38, SWE 18.92, BRA 13.51, USA 9.46, CHN 8.11, CUB 6.76, FIN 5.41, FXX 4.05 |
| ■■■■□■■■■■■■■■■■■■■■■■■■■■■■■■■■□□□□■■■□■■■ |  |  |  |  |

* Worldwide distribution is reported for regions with ≥3% of a given SITs as compared to their total number in the SITVIT2 database. The definition of macro-geographical regions and sub-regions (http://unstats.un.org/unsd/methods/m49/m49regin.htm) is according to the United Nations; Regions: AFRI (Africa), AMER (Americas), ASIA (Asia), EURO (Europe), and OCE (Oceania), subdivided in: E (Eastern), M (Middle), C (Central), N (Northern), S (Southern), SE (South-Eastern), and W (Western). Furthermore, CARIB (Caribbean) belongs to Americas, while Oceania is subdivided in 4 sub-regions, AUST (Australasia), MEL (Melanesia), MIC (Micronesia), and POLY (Polynesia). Note that in our classification scheme, Russia has been attributed a new sub-region by itself (Northern Asia) instead of including it among rest of the Eastern Europe. It reflects its geographical localization as well as due to the similarity of specific TB genotypes circulating in Russia (a majority of Beijing genotypes) with those prevalent in Central, Eastern and South-Eastern Asia.

** The three letter country codes are according to http://en.wikipedia.org/wiki/ISO_3166-1_alpha-3; countrywide distribution is only shown for SITs with ≥3% of a given SITs as compared to their total number in the SITVIT2 database.
