# Supplementary material for: Mycobacterium tuberculosis Strains Potentially Involved in the TB Epidemic in Sweden a Century Ago
Source: PLoS One. 2012 Oct 8;7(10):e46848. doi: 10.1371/journal.pone.0046848 (PMC3466202; doi:10.1371/journal.pone.0046848)
Supplement: Table S4 — Description of rare PGG1 spoligotype patterns in this study (n = 15 patterns containing 24 isolates), and their worldwide distribution. (DOCX) [file pone.0046848.s006.docx]

**Table S4.** Description of rare PGG1 spoligotype patterns in this study (n=15 patterns containing 24 isolates), and their worldwide distribution.

| **SIT (Clade) Octal Number** | **Number (%) in this study** | **% in study vs. database** | **Distribution in Regions with ≥ 5% of a given SITs *** | **Distribution in countries with ≥ 5% of a given SITs **** |
| --- | --- | --- | --- | --- |
| **Spoligotype Description** |  |  |  |  |
| 1 (Beijing) 000000000003771 | 3 (0.73) | 0.03 | ASIA-E 33.65, AMER-N 20.65, ASIA-SE 9.32, AFRI-S 8.49, ASIA-N 7.1 | USA 20.31, CHN 19.44, JPN 11.8, ZAF 8.49, RUS 7.1 |
| □□□□□□□□□□□□□□□□□□□□□□□□□□□□□□□□□□■■■■■■■■■ |  |  |  |  |
| 691 (BOV_1) 676573777777600 | 4 (0.98) | 0.55 | EURO-S 71.76, AMER-S 12.81 | ESP 71.76, ARG 9.23 |
| ■■□■■■■■□■□■■■■□■■■■■■■■■■■■■■■■■■■■■■□□□□□ |  |  |  |  |
| 1185 (BOV_1) 676673777777600 | 1 (0.24) | 33.33 | EURO-N 66.67, EURO-W 33.33 | SWE 66.67, FXX 33.33 |
| ■■□■■■■■□■■□■■■□■■■■■■■■■■■■■■■■■■■■■■□□□□□ |  |  |  |  |
| 3170* (BOV_1) 676773776777600 | 3 (0.73) | 75 | EURO-N 100 | SWE 100 |
| ■■□■■■■■□■■■■■■□■■■■■■■■■■□■■■■■■■■■■■□□□□□ |  |  |  |  |
| 3184* (BOV_1) 676773747777600 | 1 (0.24) | 50 | EURO-N 100 | SWE 100 |
| ■■□■■■■■□■■■■■■□■■■■■■□□■■■■■■■■■■■■■■□□□□□ |  |  |  |  |
| Orphan (BOV_1) 676771777777600 | 1 (0.24) | 100 | EURO-N 100 | SWE 100 |
| ■■□■■■■■□■■■■■■□□■■■■■■■■■■■■■■■■■■■■■□□□□□ |  |  |  |  |
| Orphan (BOV_LIKE) 676761037777600 | 1 (0.24) | 100 | EURO-N 100 | SWE 100 |
| ■■□■■■■■□■■■■■□□□■□□□□■■■■■■■■■■■■■■■■□□□□□ |  |  |  |  |
| 26 (CAS1-Delhi) 703777740003771 | 1 (0.24) | 0.08 | ASIA-S 52.71, AMER-N 16.95, EURO-W 6.58, ASIA-W 6.5, AFRI-E 5.5 | IND 30.11, USA 16.95, PAK 11.15, SAU 6.04, BGD 5.8 |
| ■■■□□□□■■■■■■■■■■■■■■■□□□□□□□□□□□□■■■■■■■■■ |  |  |  |  |
| 356 (CAS1-Delhi) 703777600001771 | 1 (0.24) | 7.14 | AFRI-S 35.71, EURO-N 21.43, ASIA-S 14.29, AFRI-N 14.29, ASIA-W 7.14, AMER-N 7.14 | ZAF 35.71, IND 14.29, SDN 14.29, GBR 14.29, USA 7.14, SWE 7.14, SAU 7.14 |
| ■■■□□□□■■■■■■■■■■■■■□□□□□□□□□□□□□□□■■■■■■■■ |  |  |  |  |
| 485 (CAS1-Delhi) 703777400003771 | 1 (0.24) | 4.35 | ASIA-S 47.83, AMER-N 26.09, ASIA-W 8.7, EURO-N 8.7 | IRN 30.43, USA 26.09, IND 8.7, SAU 8.7, PAK 8.7 |
| ■■■□□□□■■■■■■■■■■■■□□□□□□□□□□□□□□□■■■■■■■■■ |  |  |  |  |
| 19 (EAI2-Manilla) 677777477413771 | 3 (0.73) | 0.35 | AMER-N 56.93, ASIA-SE 18.82, ASIA-E 7.04 | USA 47.92, PHL 10.62, MEX 9.01 |
| ■■□■■■■■■■■■■■■■■■■□□■■■■■■■□□□□■□■■■■■■■■■ |  |  |  |  |
| 947 (EAI5) 777777777413631 | 1 (0.24) | 10 | EURO-N 40.0, ASIA-S 20.0, ASIA-SE 20.0, AMER-N 20.0 | SWE 30.0, IND 20.0, USA 20.0, DNK 10.0, THA 10.0, MYS 10.0 |
| ■■■■■■■■■■■■■■■■■■■■■■■■■■■■□□□□■□■■■■□□■■■ |  |  |  |  |
| 54 (Manu2) 777777777763771 | 1 (0.24) | 0.45 | ASIA-E 16.74, AFRI-N 16.29, ASIA-S 13.57, ASIA-W 11.76, AMER-N 10.86, AFRI-S 7.24 | CHN 16.74, EGY 15.38, IND 12.67, SAU 9.05, USA 9.05, ZAF 7.24 |
| ■■■■■■■■■■■■■■■■■■■■■■■■■■■■■■■■□□■■■■■■■■■ |  |  |  |  |
| 100 (Manu1) 777777777773771 | 1 (0.24) | 1.32 | ASIA-S 39.47, ASIA-W 23.68, AMER-N 14.47, EURO-N 9.21 | IND 39.47, SAU 23.68, USA 9.21, GBR 7.89 |
| ■■■■■■■■■■■■■■■■■■■■■■■■■■■■■■■■■□■■■■■■■■■ |  |  |  |  |
| 523 (Manu_ancestor) 777777777777771 | 1 (0.24) | 2.27 | ASIA-E 29.55, ASIA-SE 18.18, AMER-N 11.36, ASIA-W 9.09, AFRI-W 9.09, EURO-W 9.09 | USA 11.36, SAU 9.09, CHN 9.09, JPN 9.09, KOR 6.82, MYS 6.82, NGA 6.82 |
| ■■■■■■■■■■■■■■■■■■■■■■■■■■■■■■■■■■■■■■■■■■■ |  |  |  |  |

* Worldwide distribution is reported for regions with ≥5% of a given SITs as compared to their total number in the SITVIT2 database. The definition of macro-geographical regions and sub-regions (<http://unstats.un.org/unsd/methods/m49/m49regin.htm>) is according to the United Nations; Regions: AFRI (Africa), AMER (Americas), ASIA (Asia), EURO (Europe), and OCE (Oceania), subdivided in: E (Eastern), M (Middle), C (Central), N (Northern), S (Southern), SE (South-Eastern), and W (Western). Furthermore, CARIB (Caribbean) belongs to Americas, while Oceania is subdivided in 4 sub-regions, AUST (Australasia), MEL (Melanesia), MIC (Micronesia), and POLY (Polynesia). Note that in our classification scheme, Russia has been attributed a new sub-region by itself (Northern Asia) instead of including it among rest of the Eastern Europe. It reflects its geographical localization as well as due to the similarity of specific TB genotypes circulating in Russia (a majority of Beijing genotypes) with those prevalent in Central, Eastern and South-Eastern Asia.

** The three letter country codes are according to <http://en.wikipedia.org/wiki/ISO_3166-1_alpha-3>; countrywide distribution is only shown for SITs with ≥5% of a given SITs as compared to their total number in the SITVIT2 database.
